# Age-sex differences in Alzheimer’s and related dementias burden and risk factors in east and Southeast Asia: results from the 2021 GBD study

**DOI:** 10.3389/fnagi.2025.1562148

**Published:** 2025-06-27

**Authors:** Tengyu Zhao, Pengyu Pan, Yuhan Zhou, Xinyue Zhang, Quan Li, Yanyan Zhou

**Affiliations:** ^1^School of Basic Medicine, Heilongjiang University of Chinese Medicine, Harbin, China; ^2^Key Laboratory of Basic Theory Research on Traditional Chinese Medicine, Heilongjiang University of Chinese Medicine, Harbin, China

**Keywords:** GBD (2021) database, Alzheimer’s disease and related dementias, burden of disease, risk factor, age-sex differences, East and Southeast Asia

## Abstract

**Background:**

Alzheimer’s disease and related dementias (ADRD) significant global public health challenges, leading to severe disability in patients and placing a heavy burden on caregivers. However, epidemiological studies focusing on ADRD in specific regions remain limited. This study aims to comprehensively analyze and describe the current status and changing trends of ADRD in Non-High-income East Asia (NHIEA), Non-High-income Southeast Asia (NHISEA), and High-income Asia Pacific (HIAP), providing more detailed real-world data to inform policymaking.

**Methods:**

The data for ADRD used in this study were extracted from the 2021 Global Burden of Disease (GBD) database. We employed three major indicators of disease burden—prevalence, incidence, and years lived with disability (YLD)—and explored associated risk factors, further analyzing trends by age and sex. The results are presented as mean values with 95% uncertainty intervals (UIs). Additionally, we explored the differences between NHIEA, NHISEA, HIAP and other regions, as well as the potential associations between the disease burden of Alzheimer’s and other dementias and socioeconomic factors.

**Results:**

The findings indicate that the burden of dementia is rising in East and Southeast Asia, with women showing a higher burden across all indicators. Notably, in NHIEA, particularly in China, the burden of dementia has increased with the rising Social Demographic Index (SDI). China experienced a 27.3% increase in Alzheimer’s disease and other dementia ASYRs from 1990 to 2021, with a sharp 7.6% annual surge in 2021 alone, outpacing regional averages. Gender analysis revealed that women bear a disproportionate burden of Alzheimer’s disease and related dementias, especially after menopause, when the risk increases significantly. The study also identified smoking, high blood sugar, and high body mass index as important risk factors affecting the disease burden. The contribution of these risk factors varies across regions, genders, and age groups.

**Conclusion:**

The health burden of ADRD remains substantial, with distinct patterns observed across NHIEA, NHISEA, and HIAP, including regional variations in gender, age, and risk factors. These findings highlight the need for tailored approaches to allocate healthcare resources and implement appropriate control measures based on the specific conditions of each region to address this growing public health challenge. Future research should prioritize comparative analyses across continents and within regions to inform the development of more region-specific prevention strategies for ADRD.

## 1 Introduction

China, as one of the leading country in Asia and the world, has many well-known education programs and long-term daily strategic practices to enhance various brain functions involving learning, memory, neuroplasticity and executive perspectives, such as chess playing, abacus training and calligraphy for Chinese people starting from even childhood to youth stage (Yongxia, 2023). Alzheimer’s disease and related dementias (ADRD) are a group of progressive neurodegenerative diseases, primarily dominated by Alzheimer’s disease, which mainly affect memory, cognitive function, and behavior. Over time, these conditions impair the ability of patients to carry out daily activities (Kukull et al., 1992). With the global aging population and increasing life expectancy, the incidence of Alzheimer’s disease and other forms of dementia continues to rise, resulting in a significant public health challenge that impacts individuals, families, and healthcare systems alike (Pickett and Brayne, 2019). According to a 2019 report, dementia is the seventh leading cause of death worldwide, accounting for 17.3% (1.6 million) of all deaths from neurological diseases. Among people aged 70 and older, it is the fourth leading cause of death (van der Flier et al., 2023). By 2019, the global number of people living with ADRD had risen to 51.62 million, and it is projected to increase to 152.8 million by 2050 (Nichols et al., 2022). Moreover, The same report also indicates that East Asia has the highest ASPR of Alzheimer’s and related dementias (ASPR 781.43/100,000), with Southeast Asia also experiencing a steady rise in the burden of these diseases. This suggests that these regions are facing a greater social and healthcare burden due to Alzheimer’s disease and other dementias (Li et al., 2022).

Currently, there is no cure or effective treatment to slow the progression of ADRD. However, multiple factors—such as smoking, obesity, alcohol consumption, and cardiovascular conditions, including microvascular inflammation, ischemia, and white matter demyelination—have been identified as significant risk factors for cognitive decline and Alzheimer’s disease (AD) in older adults (Yongxia, 2016). Therefore, identifying modifiable risk factors to reduce the incidence of the disease, delay its onset, or mitigate its impact has become increasingly important. Existing research has identified several major risk factors for ADRD, including low educational attainment, hypertension, hearing loss, smoking, obesity, depression, lack of physical activity, diabetes, air pollution, and social isolation. Interventions targeting these risk factors could prevent or delay up to 40% of dementia cases (Livingston et al., 2020). Beyond epidemiology, geroscience research shows that modifiable lifestyle interventions—structured physical activity, Mediterranean-style diets, and cognitive training—can delay AD onset and extend healthspan. A recent meta-analysis reported up to 40% reduction in dementia incidence following comprehensive wellness programs (Gao et al., 2024), underscoring the value of integrating anti-aging strategies into primary care.

Although numerous global and cross-regional studies have examined the burden of Alzheimer’s disease and related dementias (ADRD), region-specific analyses remain limited. A recent GBD-based study (Guo et al., 2025)provided a broad overview of the disease burden, associated risk factors, and future projections of ADRD in Asia. This important work highlighted that over the next 30 years, the burden of ADRD in Asia is expected to increase significantly, building upon an already substantial baseline. However, the analysis of risk factors in that study was relatively general, lacking stratification by socio-demographic index (SDI), age, and sex. These dimensions are critical for understanding vulnerable subpopulations and tailoring effective interventions. Therefore, there remains a need for more detailed research focused on specific regions, such as East Asia and Southeast Asia, to inform targeted strategies for burden reduction. These regions are characterized by distinct racial, physiological, social, and cultural factors, which contribute to health issues that differ from those in other parts of the world. Both East Asia and Southeast Asia are currently facing the dual challenges of an aging population and rapid urbanization, which not only intensify the disease burden but also introduce new health risks. Therefore, this study will focus on analyzing the incidence, prevalence, and disability-adjusted life years (DALY) of Alzheimer’s and related dementias in East and Southeast Asia, as well as exploring the associated risk factors. The findings of this research will provide critical theoretical support for interventions aimed at reducing the disease burden in these regions and contribute to the development of more targeted public health strategies. Furthermore, it will lay the groundwork for future research in this area.

Although this review focuses on East and Southeast Asia, ADRD is not unique to these regions but is observed worldwide. Many countries share a similar disease load of ADRD, comparable to that in East and Southeast Asia. According to the GBD 2021 estimates, the highest ASPRs for ADRD were found in High income Asia Pacific (811 per 100,000), Western Europe (802 per 100,000), and High-income North America (785 per 100,000), with no significant differences observed between sexes (Wang, 2025). Moreover, the incidence rate in both East Asia and Germany peaks at over 2,500 cases per 100,000 population in individuals aged 80–84 and 85–89 years (Amiri et al., 2025). Nevertheless, detailed comparisons of age, sex, and risk-factor contributions in these diverse settings remain limited and warrant further region-specific analyses.

## 2 Methods

### 2.1 Data source

We extracted data on the disease prevalence, incidence, YLD and risk factors for Alzheimer’s disease (AD) and other dementias in East Asia and Southeast Asia from the Global Burden of Disease (GBD) results tool.^1^ GBD 2021 provides comprehensive global health data for 371 countries and regions, covering 204 diseases and injuries, including 21 countries with subnational data (GBD, 2021a). The data sources include verbal autopsies, household surveys, vital registration systems, specific disease registries, healthcare utilization records, and other sources. Additionally, GBD 2021 follows the guidelines set out in the “Accurate and Transparent Health Estimation Reporting Standards.” Previous studies have extensively documented the comprehensive data sources and detailed analytical methods used in GBD 2021 (GBD 2021 Risk Factors Collaborators, 2024). The major pathological causes of dementia include AD, vascular dementia, Lewy body dementia, and frontotemporal dementia (Elahi and Miller, 2017). AD is the most common type of dementia. ICD-10 assigns different disease codes for AD and other dementias in the GBD database, including F00, F01, F02, F03, G30, and G31 (GBD, 2021b). The comparative risk assessment framework of GBD identifies three primary risks for AD and other dementias: high fasting plasma glucose, high body mass index, and smoking. GBD 2021 defines high FPG as any level above the theoretical minimum risk exposure level (TMREL) of 4.9–5.3 mmol/L, high BMI as any BMI greater than 20 to 23 kg/m (Kukull et al., 1992), and smokers as individuals who currently use any tobacco products daily or occasionally (GBD, 2021c,d,e). Based on these datasets, regions are categorized into three distinct groups according to their geographic and economic conditions: Non-High-income East Asia(NHIEA), Non-High-income Southeast Asia(NHISEA)and High-income Asia Pacific(HIAP).

### 2.2 Measures of disease burden

This study uses prevalence, incidence, and Years Lived with Disability (YLD) as the primary indicators to assess the current status and trends of the disease burden of ADRD in NHIEA, NHISEA, and HIAP region. Prevalence refers to the actual number of cases of a specific disease or injury in the general population. Incidence represents the number of newly diagnosed cases over a defined period. YLD represents the years of life lived with disability due to short- or long-term health impairment, weighted by severity according to disability weights derived from public surveys. Both absolute numbers and rates are used to describe the above three indicators, facilitating a multidimensional understanding of the disease burden of ADRD. Point estimates and 95% confidence intervals (95% CI) for these indicators were calculated based on 1,000 draw values during the GBD modeling process (GBD 2019 Diseases and Injuries Collaborators, 2020). All indicators are stratified by year, region, age, and sex.

### 2.3 Statistical analysis and results presentation

GBD data are provided as aggregated statistics without individual sample sizes or measures of dispersion (e.g., standard deviation [SD] or standard error [SE]); therefore, our analyses adhere to GBD reporting standards. Our research team first analyzed and compared the age-standardized prevalence rate (ASPR), age-standardized incidence rate (ASIR), and age-standardized YLD rate (ASYR) for ADRD in the NHIEA, NHISEA, and HIAP regions in 2021, as well as in specific countries and regions within these areas. We then focused on assessing the absolute numbers and rates of these indicators across different age groups in the three regions, including the sex ratio of prevalence, incidence, and YLD. Additionally, we evaluated the burden of risk factors associated across different age-sex groups. Finally, we compared the differences in the prevalence, incidence, YLD and risk factors of ADRD between the NHIEA, NHISEA, and HIAP, as well as individual countries and globally. Additionally, we explored the potential association between the Socio-Demographic Index (SDI) and the ASYR for ADRD.

Statistical analyses and visualizations were performed using R (version 4.3.1). In addition, AI-assisted tools, such as OpenAI ChatGPT and Grammarly, were used for tasks like text translation, clarity refinement, and grammar correction. These tools were employed to enhance precision and grammatical accuracy, not to generate content.

## 3 Result

### 3.1 NHIEA, NHISEA, and HIAP

According to Table 1, in 2021, there were 17,414,173 existing cases (95% UI: 14,854,023 to 20,142,130), 2,988,724 newly diagnosed cases (95% UI: 2,569,166 to 3,434,391), and 3,548,572 YLD (95% UI: 2,454,542 to 4,745,452) attributed to Alzheimer’s disease and other dementias in NHIEA.

**Table 1:** The absolute numbers, age-standardized rates, and annual change rates of age-standardized rates of prevalence, incidence, and YLDs of Alzheimer’s disease and other in Non-High-income East Asia, Non-High-income Southeast Asia and High-income Asia Pacific.

Alzheimer’s disease and other dementias	Prevalence (95% UI)			Incidence (95% UI)			YLDs (95% UI)		
Country/Region	Number (2021)	ASPR (2021)	Change rate of ASPR (1990–2021)	Number (2021)	ASIR (2021)	Change rate of ASIR (1990–2021)	Number (2021)	ASYR (2021)	Change rate of ASYR (1990–2021)
Non-High-income East Asia	17,414,173 (14,854,023, 20,142,130)	887.95 (759.95, 1027.48)	27.35% (23.82, 30.36%)	2,988,724 (2,569,166, 3,434,391)	149.61 (129.58, 171.14)	24.38% (20.71, 27.38%)	3,548,572 (2,454,542, 4,745,452)	183.01 (126.4, 242.99)	26.56% (23.15, 29.59%)
People’s Republic of China	16,990,827 (14,488,494, 19,672,741)	900.82 (770.92, 1043.22)	28.11% (24.47, 31.11%)	2,914,112 (2,504,728, 3,350,743)	204.82 (176.05, 235.51)	25.07% (21.27, 28.17%)	3,460,324 (2,394,267, 4,632,167)	185.63 (127.98, 246.72)	27.34% (23.93, 30.44%)
Democratic People’s Republic of Korea	177,068 (151,286, 204,071)	618.92 (529.05, 710.41)	−1.49% (−7.39,4.26%)	31,408 (26,865, 36,668)	108.5 (93.89, 125.37)	−2.16% (−6.24, 1.72%)	36,771 (24,931, 48,631)	130.83 (89.99, 172.79)	−1.98% (−6.46, 2.77%)
Taiwan (Province of China)	246,278 (209,401, 276,216)	555.31 (472.14, 624.76)	9.39% (0.94, 20.32%)	43,204 (36,980, 48,565)	98.1 (83.69, 110.2)	7.55% (−0.33,17.97%)	51,478 (36,251, 68,152)	115.32 (80.92, 151.93)	9.32% (−0.14, 20.88%)
Non-High-income Southeast Asia	3,387,799 (2,922,771, 3,857,741)	644.38 (560.58, 737.69)	−4.54% (−5.83, −3.37%)	578,242 (505,927, 658,513)	110.07 (96.11, 125.72)	−4.16% (−5.28, 3.03%)	681,425 (468,311, 903,801)	131.35 (90.24, 173.11)	−4.31% (−5.83, −2.83%)
Kingdom of Cambodia	59,413 (50,607, 68,052)	658.61 (568.01, 754.43)	−3.6% (−7.02, −0.19%)	10,162 (8,838, 11,662)	112.21 (97.73, 128.37)	−3.33% (−6.36, −0.32%)	11,825 (8,045, 16,025)	133.84 (92.28, 177.25)	−3.1% (−7.66, 1.66%)
Republic of Indonesia	1,109,364 (953,356, 1,273,166)	662.13 (571.11, 761.15)	−2.44% (−3.98, −0.99%)	189,722 (164,142, 216,604)	113.31 (98.38, 129.52)	−2.29% (−3.61, −1.07%)	220,375 (149,845, 293,427)	134.72 (92.55, 178.47)	−2.05% (−3.89, −0.2%)
Lao People’s Democratic Republic	21,888 (18,988, 24,981)	655.85 (567.01, 749.46)	−2.74% (−5.93, 1.42%)	3,764 (3,280, 4,290)	111.82 (97.43, 127.89)	−2.13% (−5.23, 1.05%)	4,405 (3,061, 5,905)	134.78 (93.21, 178.82)	−3.02% (−7.05, −2.11%)
Malaysia	153,807 (130,889, 175,275)	657.55 (561.66, 750.29)	−5.46% (−8.77, −2.32%)	26,046 (22,443, 29,858)	111.29 (95.99, 127.46)	−4.17% (−7.47, −1.17%)	30,596 (20,862, 40,952)	132.38 (90.72, 175.45)	−6.08% (−10.39, −1.93%)
Republic of Maldives	1,794 (1,545, 2,056)	667.3 (572.03, 764.23)	3.08% (−0.34, 6.59%)	309(269, 355)	113.22 (98.24, 129.67)	3.07% (−0.08, 6.34%)	362 (251, 479)	135.81 (94.36, 178.83)	4.37% (−0.01, 9.43%)
Republic of the Union of Myanmar	257,641 (222,039, 292,332)	663.53 (571.48, 757.76)	−7.91% (−10.88, −4.38%)	44,028 (38,392, 49,952)	113.09 (99.06, 128.82)	−7.47% (−10.23, −4.32%)	51,653 (35,564, 68,865)	134.77 (92.92, 178.4)	−7.68% (−11.55, −3.08%)
Republic of the Philippines	419,363 (364,503, 477,877)	662.94 (575.83,762.08)	−4.2% (−5, −3.48%)	72,092 (63,052, 81,980)	113.91 (99.74, 130)	−4.3% (−5.03, −3.68%)	83,965 (57,047, 111,296)	134.71 (92.57, 178.57)	−2.83% (−4.11, −1.67%)
Democratic Socialist Republic of Sri Lanka	152,889 (129,859, 175,784)	634.13 (545.74,727.67)	−3.76% (−6.84, −0.14%)	25,786 (22,299, 29,882)	107.46 (93.3, 123.54)	−3.44% (−6.34, 0.07%)	30,325 (20,848, 40,240)	127.28 (88.33, 167.26)	−4.21% (−8.27, 0.14%)
Kingdom of Thailand	659,437 (571,371, 751,935)	603.22 (522.58,689.6)	0.31% (−4.12, 4.61%)	112,595 (98,738, 127,875)	103.27 (90.64, 117.58)	0.75% (−2.97, 4.45%)	135,193 (93,176, 177,810)	123.5 (85.05, 162.68)	0.54% (−4.55, 5.24%)
Democratic Republic of Timor-Leste	4,508 (3,824, 5,177)	664.45 (572.75,759.71)	−6.63% (−9.59, −3.51%)	768 (666, 888)	113.2 (98.46, 129.74)	−5.96% (−8.91, −3.03%)	888 (614, 1,183)	133.5 (93.05, 174.12)	−6.67% (−10.74, −2.41%)
Socialist Republic of Viet Nam	531,198 (453,586, 601,711)	649.42 (556.89,744.39)	−5.8% (−9.07, −2.57%)	90,172 (78,824, 102,840)	110.09 (96.21, 126.57)	−5.54% (−8.65, −2.59%)	108,523 (74,047, 144,441)	134.08 (92.23, 176.55)	−5.68% (−9.96, −1.47%)
Mauritius	11,136 (9,549, 12,616)	657.17 (567.03,748.3)	−0.94% (−4.55, 2.61%)	1883 (1,629, 2,139)	111.73 (96.55, 127.55)	−0.56% (−3.88, 2.69%)	2,237 (1,554, 2,960)	132.58 (91.94, 173.2)	−2.49% (−7.12, 1.86%)
Seychelles	636 (543, 732)	643.49 (544.72,741.31)	−3.66% (−6.99, −0.02%)	108 (94, 124)	109.25 (94.47, 126.6)	−3.43% (−6.71, −0.22%)	127 (88, 168)	130.16 (89.69, 172.41)	−5.91% (−10.11, −1.58%)
High-income Asia Pacific	4,109,524 (3,547,341, 4,692,562)	684.82 (596.97,780.11)	4.07% (1.9, 6.14%)	701,760 (614,636, 802,668)	118.62 (103.43, 135)	1.44% (−0.45, 3.33%)	876,386 (597,026, 1,168,904)	143.1 (97.66, 190.15)	4.15% (1.96, 6.24%)
Brunei Darussalam	1,331 (1,141, 1,519)	578.96 (496.58, 663.75)	−0.4% (−4.07, 3.82%)	237 (205, 273)	101.56 (88.37, 116.86)	−0.35% (−4.11, 3.39%)	264 (181, 349)	118.75 (81.04, 157.27)	−0.43% (−5.14, 5.18%)
Japan	3,367,358 (2,893,951, 3,872,084)	674.1 (584.78, 770.24)	3.76% (1.84, 5.53%)	576,270 (503,457, 662,794)	117.23 (102.05, 133.68)	1.22% (−0.42, 2.83%)	723,485 (490,874, 977,487)	141.25 (96.29, 188.04)	4.03% (1.98, 5.99%)
Republic of Korea	697,045 (609,566, 789,739)	739.81 (647.7, 836.31)	−1.49% (−7.39, 4.26%)	117,489 (102,910, 133,127)	124.63 (109.44, 140.89)	−2.41% (−8.06, 3.06%)	461,797 (218,816, 928,411)	152.76 (104.41, 202.48)	−2.08% (−8.18, 4.73%)
Republic of Singapore	43,791 (39,004, 48,432)	534.49 (474.45, 589.82)	3.57% (−1.61, 9.96%)	7,764 (6,988, 8,584)	94.88 (85.54, 104.85)	3.63% (−1.27, 9.95%)	9,230 (6,524, 12,061)	112.64 (79.44, 147.06)	4.04% (−1.95, 11.17%)

For NHISEA, a total of 3,387,799 existing Alzheimer’s disease and other dementias cases (95% UI: 2,922,771 to 3,857,741), 578,242 newly diagnosed Alzheimer’s disease and other dementias cases (95% UI: 505,927 to 658,513), and 681,425 years lived with Alzheimer’s disease and other dementias related disability (95% UI: 468,311 to 903,801) were reported in 2021.

For HIAP, a total of 4,109,524 existing Alzheimer’s disease and other dementias cases (95% UI: 3,547,341 to 4,692,562), 701,760 newly diagnosed Alzheimer’s disease and other dementias cases (95% UI: 614,636 to 802,668), and 876,386 years lived with Alzheimer’s disease and other dementias related disability (95% UI: 597,026 to 1,168,904) were reported in 2021.

### 3.2 National and regional levels

As shown in Table 1, in 2021, the national and regional ASPRs of Alzheimer’s disease and other dementias ranged from 534.49 to 900.82 cases per 100,000 population, among which People’s Republic of China had the highest ASPR of Alzheimer’s disease and other dementias [900.82 (95% UI: 770.92 to 1043.22)] and Republic of Singapore had the lowest ASPR of Alzheimer’s disease and other dementias [534.49 (95% UI: 474.45 to 589.82)]. Moreover, the annual percent changes in the ASPR of Alzheimer’s disease and other dementias from 1990 to 2021 ranged from −7.91 to 28.11%. People’s Republic of China had the greatest percent change in the ASPR of Alzheimer’s disease and other dementias [28.11% (95% UI: 24.47 to 31.11%)], and Republic of the Union of Myanmar had the smallest percent change in the ASPR of Alzheimer’s disease and other dementias [−7.91% (95% UI: −10.88% to −4.38%)].

In terms of incidence, the national and regional ASIRs of Alzheimer’s disease and other dementias ranged from 94.88 to 204.82 cases per 100,000 population in 2021. The highest ASIR of Alzheimer’s disease and other dementias was found in People’s Republic of China [204.82 (95% UI: 176.05 to 235.51)], and the lowest ASIR of lzheimer’s disease was found in Republic of Singapore [94.88 (95% UI: 85.54 to 104.85)]. Moreover, the annual percent changes in the ASIR of Alzheimer’s disease and other dementias from 1990 to 2021 ranged from −7.47 to 25.07%. China had the greatest percent change in the ASIR of Alzheimer’s disease and other dementias [25.07% (95% UI: 21.27 to 28.17%)], and Republic of the Union of Myanmar had the smallest percent change in the ASIR of Alzheimer’s disease and other dementias [−7.47% (95% UI: −10.23% to −4.32%)].

In regard to YLD, in 2021, the national and regional ASYRs of Alzheimer’s disease and other dementias ranged from 112.64 to 185.63 cases per 100,000 population. People’s Republic of China had the highest ASYR of Alzheimer’s disease and other dementias [185.63 (95% UI: 127.98 to 246.72)]. However, the lowest ASYR of Alzheimer’s disease and other dementias was observed in the Republic of Singapore [112.64 (95% UI: 79.44 to 147.06)]. Moreover, the annual percent changes in the ASYR of Alzheimer’s disease and other dementias from 1990 to 2021 ranged from −7.68 to 27.34%. The greatest percent change in the ASYR of Alzheimer’s disease and other dementias was found in People’s Republic of China [27.34% (95% UI: 23.93 to 30.44%)], and the smallest percent change in the ASYR of Alzheimer’s disease and other dementias was found in Republic of the Union of Myanmar [−7.68% (95% UI: −11.55% to−3.08%)].

### 3.3 Age and sex patterns

**Figure 1:**
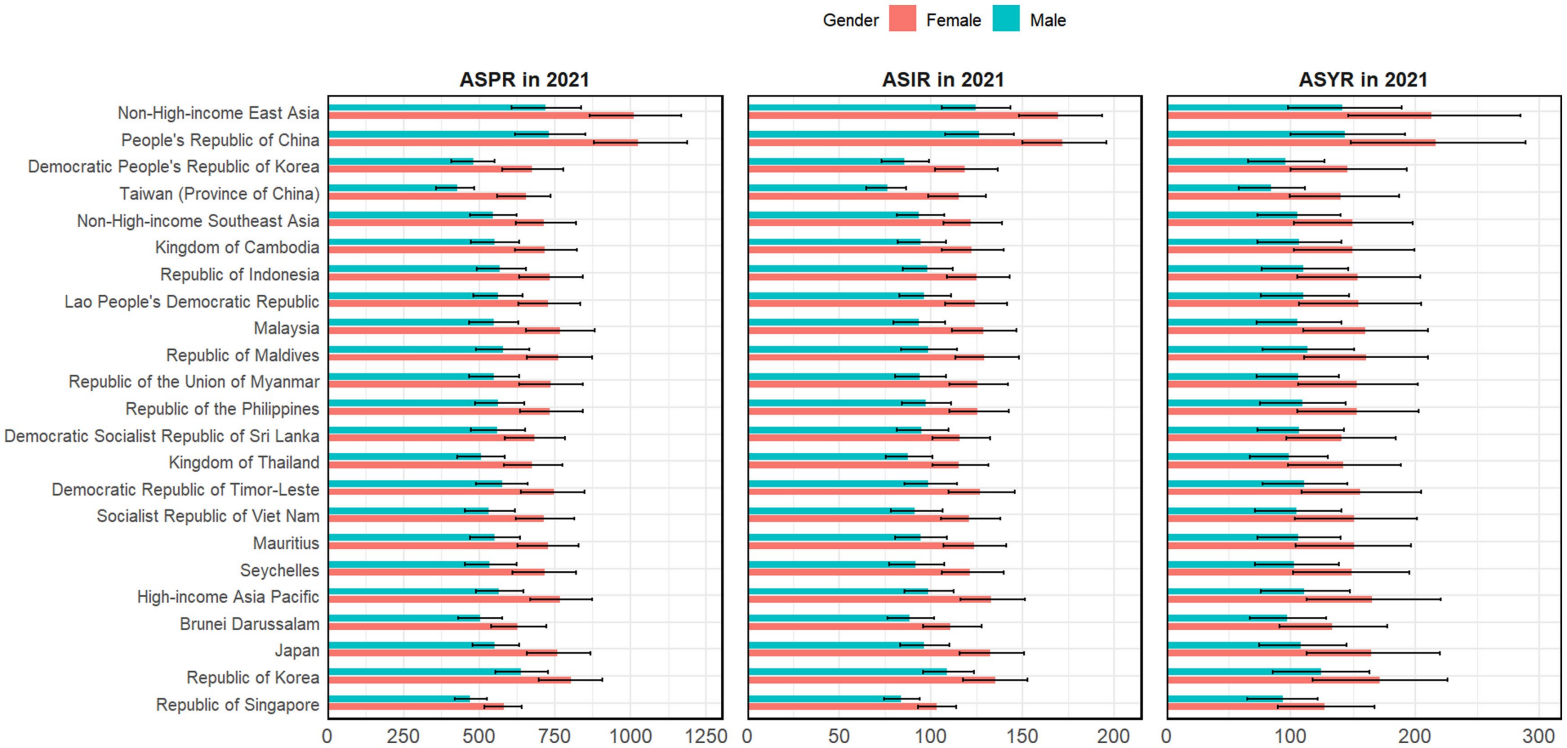
The age-standardized rates of prevalence, incidence, and YLDs of Alzheimer’s disease and other dementias in specific countries and regions in East and Southeast Asia in 2021.

In general, Alzheimer’s disease and other dementias were found to be more prevalent and disabling among females living in East and Southeast Asia. When analysed by specific countries and regions, women in People’s Republic of China had the highest age-standardized rates of all three main indicators of Alzheimer’s disease and other dementias burden (Figure 1; Supplementary Tables S1–S3).

**Figure 2:**
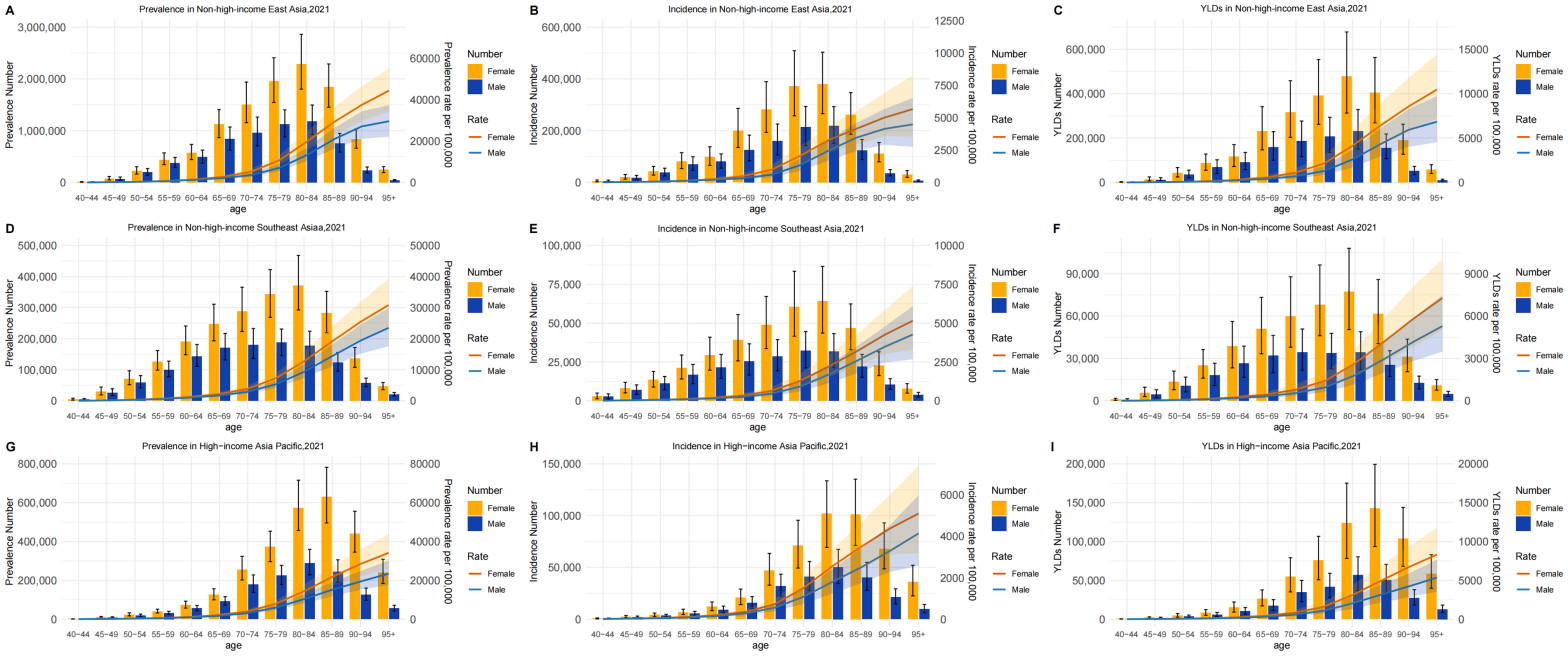
The absolute numbers and rates of prevalence, incidence, and YLDs of Alzheimer’s disease and other dementias by gender and age in EA, SEA and HIAP in 2021.

In 2021, the prevalence, incidence, and years lived with disability (YLD) rate and numbers for Alzheimer’s disease and other dementias showed distinct patterns by age, sex, and region. Regarding the prevalence numbers, In NHIEA, both males and females peaked in the 80–84 age group (Figure 2A; Supplementary Table S4). In NHISEA, males peaked in the 75–79 age group, and females peaked in the 80–84 age group (Figure 2D; Supplementary Tables S5). In HIAP, males peaked in the 80–84 age group, and females peaked in the 85–89 age group (Figure 2E; Supplementary Table S6).

Regarding the incidence numbers, In NHIEA, both males and females peaked in the 80–84 age group (Figure 2B; Supplementary Table S7). In NHISEA, males peaked in the 75–79 age group, and females in the 80–84 age group (Figure 2E; Supplementary Table S8). In HIAP, both sexes peaked in the 80–84 age group (Figure 2H; Supplementary Table S9).

Regarding YLD numbers, In NHIEA, both males and females peaked in the 80–84 age group (Figure 2C; Supplementary Table S10). In NHISEA, both males and females peaked in the 80–84 age group (Figure 2F; Supplementary Table S11). In HIAP, males peaked in the 80–84 age group, while females peaked in the 85–89 age group (Figure 2I; Supplementary Table S12).

In 2021, compared to 1990, for the three indicators, the male-to-female ratio in NHIEA exhibited a marked decrease in the 60–75 age group, followed by a rebound after 75 years, and then declined again after 90 years. Notably, for YLDs, the upward trend began earlier (at 75 years) and was more pronounced, with the subsequent decline also occurring earlier than in 1990.

**Figure 3:**
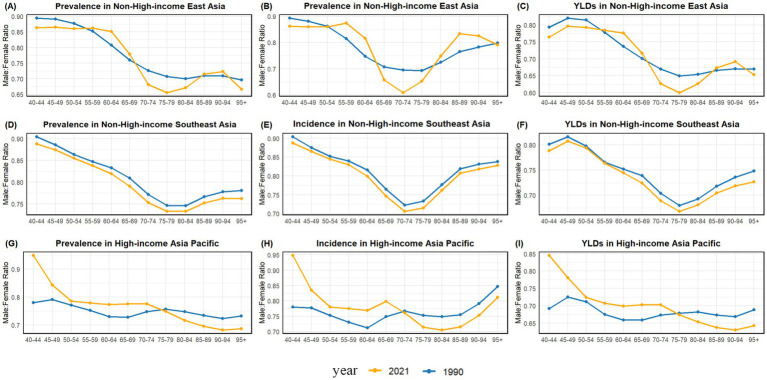
The ratios of male to female in prevalence, incidence, and YLDs of Alzheimer’s disease and other dementias **(A–I)** in EA, SEA, HIAP in 1990 and 2021.

In NHISEA, the changes in the male-to-female ratio with age for the three indicators in 2021 were broadly similar to those in 1990 (Figures 3D–F; Supplementary Table S14). The ratio showed a gradual decline between the ages of 40 and 70, reached a peak between 70 and 80 years, and then increased thereafter. A slight overall decline in the ratio was observed across all ages in 2021 compared to 1990.

In the HIAP region, the male-to-female ratio in 2021 displayed distinct trends across the three indicators compared to 1990. A sharp decline was evident in the 40–55 age group, with the ratio remaining relatively high before 70 or 75 years. After this age, the ratio dropped below 1990 levels (Figures 3G–I; Supplementary Table S15). Additionally, in 2021, the ratios for prevalence and YLDs gradually decreased, while the ratio for incidence increased after 80 years of age.

### 3.4 Risk factors’ contribution to disease burden

**Figure 4:**
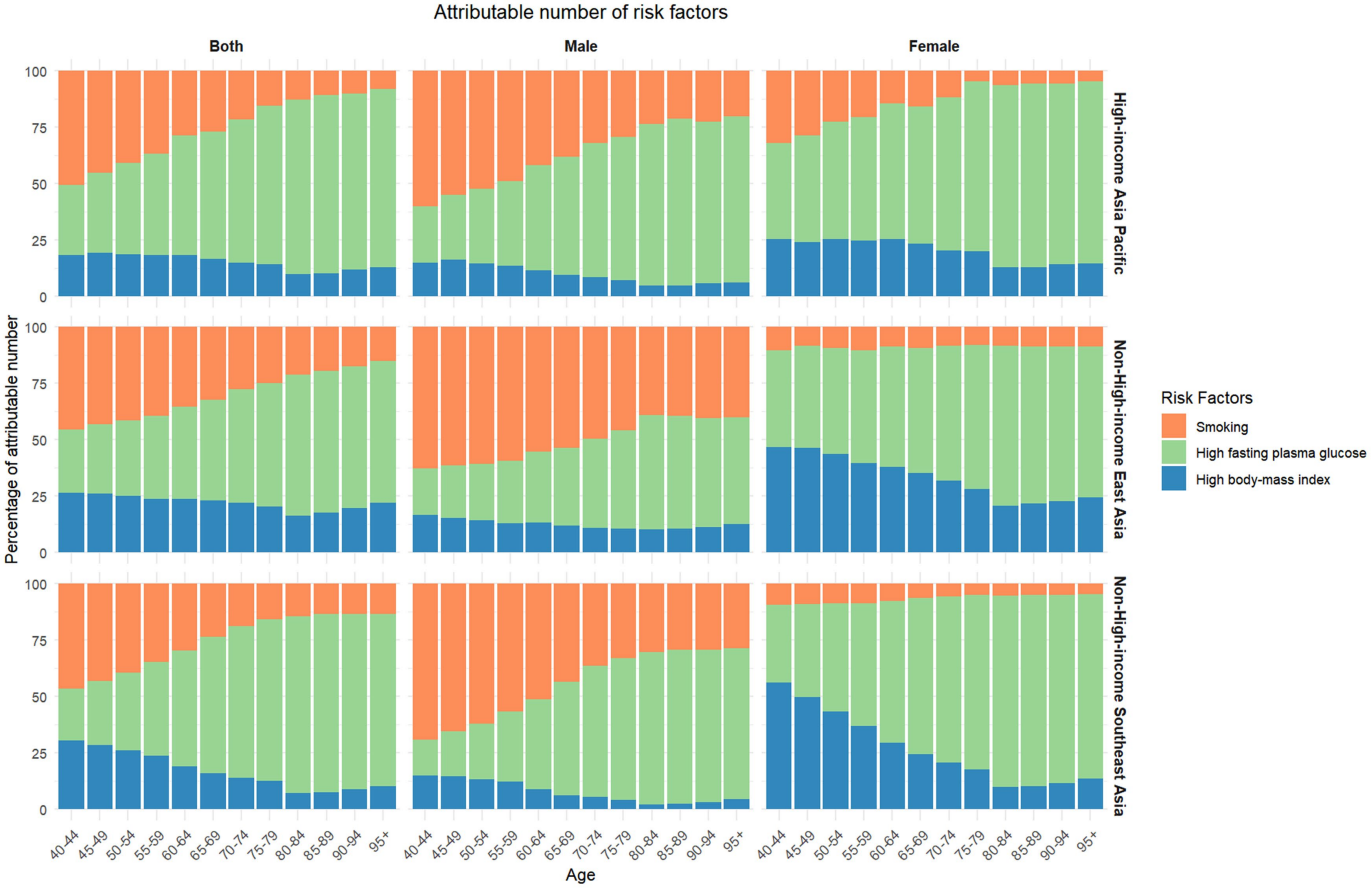
Percentage stacking chart of attributable number of different risk factors for three measures in different age and gender groups in 2021.

As shown in Figure 4 and Supplementary Tables S19–27, the trends in the attributable percentages of the three risk factors for Alzheimer’s disease and other dementias across the three regions are approximately similar. Overall, smoking is the primary risk factor in younger age groups. However, its impact declines progressively with age, while the harm associated with high fasting plasma glucose increases over time, eventually becoming the most significant risk factor in older age groups. In contrast, high body-mass index contributes less to the overall burden, and its influence decreases slightly with age. Smoking is the most important risk factor for younger males but is gradually replaced by metabolic risks as they age. For females, the impact of smoking remains the smallest among the three risk factors across all age groups. However, high body-mass index is more detrimental to younger females, especially in NHIEA and NHISEA. Furthermore, compared to other regions, males in NHIEA are the most affected by smoking, while females in HIAP are more significantly impacted by smoking.

### 3.5 Association between the SDI and ASYRs

**Figure 5:**
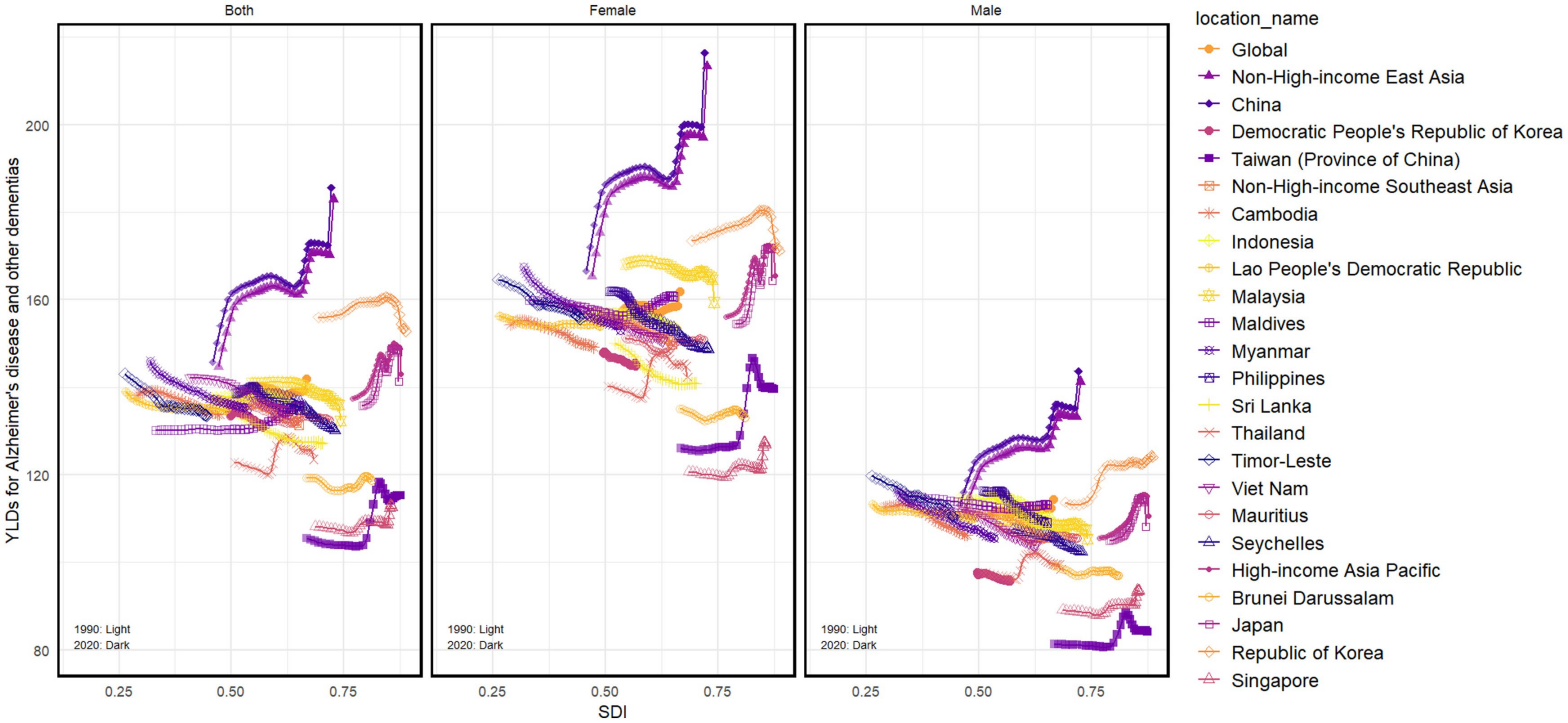
The associations between SDI and the disease burden of Alzheimer’s disease and other dementias in NHIEA, NHISEA, HIAP.

As shown in Figure 5 and Supplementary Tables S16–18, the associations between the SDI and disease ASYRs from Alzheimer’s disease and other dementias in this study were rather complicated. With the continuous increase in local SDI, the ASYRs of Alzheimer’s disease and other dementias in most countries exhibit a slight downward trend or level fluctuations, particularly in NHISEA countries and regions, such as Cambodia, Lao People’s Democratic Republic, and Myanmar, as well as in NHIEAn countries like the Democratic People’s Republic of Korea. Similar trends are -observed in some High-income Asian countries such as Brunei Darussalam. However, in countries like China, South Korea, Japan, and Singapore, the ASYRs demonstrate a marked upward trend with increasing SDI, particularly in China. The study revealed a marked upward trend in ASYRs of Alzheimer’s disease and other dementias in China, increasing from 145.78 in 1990 to 185.63 in 2021—a 27.3% rise over three decades. Notably, the year 2021 alone witnessed an accelerated surge, with ASYRs climbing from 172.45 to 185.63 (a 7.6% annual increase), surpassing the average growth rates observed in other East and South Asian countries/regions during the same period. Notably, recent years have seen a reversal of this upward trend in Japan and Taiwan (China).

When examining the gender differences in the relationship between SDI and ASYRs of ADRD, it is evident that women are disproportionately affected. Female ASYRs exhibit consistently higher levels and a more pronounced trend as SDI changes compared to males. This gender disparity is particularly prominent in countries with higher SDI levels.

## 4 Discussion

In our current work, we observed approximately 17,414,173 cases of Alzheimer’s disease and other dementias in NHIEA, 3,387,799 cases in NHISEA, and 4,109,524 cases in the HIAP region in 2021. Although individuals aged 80 to 85 were identified as the primary population affected by AD, the prevalence and incidence rates among those under 65 still accounted for a significant proportion, particularly in the 45–64 age range. This is consistent with a previous study (Withall et al., 2014). The neurodegenerative symptoms of dementia significantly disrupt the lives of younger patients, their families, and society, with profound socio-economic consequences (Kvello-Alme et al., 2020).

Additionally, women bear a greater burden of Alzheimer’s disease and other dementias than men, with this disparity increasing with age, particularly after menopause due to the loss of estrogen’s neuroprotective effects. Studies suggest this may be attributed to hormonal differences and factors related to brain development between men and women (Sohn et al., 2018). Growing evidence from brain imaging, autopsy analyses, hormone therapy, and genetic testing also indicates that AD manifests differently in terms of incidence and progression between male and female patients (Laws et al., 2016).

Notably, our analysis of age-specific male-to-female ratios reveals a significant shift during a particular age range, which may be closely related to fluctuations in estrogen levels during menopause. This phenomenon is especially evident in HIAP regions, where diagnostic and treatment capabilities are more advanced. The male-to-female of HIAP regions disparity is notably wider in the 45–50 age range, corresponding to the menopausal transition in women. Numerous studies have demonstrated the neuroprotective role of estrogen and the sharp increase in dementia risk following menopause in women (Scheyer et al., 2018; Sochocka et al., 2023).

Due to incomplete survey data and the lack of various indicators, this study focused on three key secondary risk factors—smoking, high body mass index, and high fasting plasma glucose—to better clarify their relationship with dementia. The data revealed age-related patterns in risk factor burdens, showing differences by gender and region. Smoking was identified as the primary risk factor for disease burden among men, although its impact diminished slightly with age. Similarly, a prospective cohort study demonstrated that active smoking (compared to never smoking) was associated with an increased risk of dementia. Smoking may directly link to dementia risk through its carcinogenic and harmful properties, as well as through increasing the incidence of coronary heart disease, diabetes, and other metabolic disorders, causing vasoconstriction, atherosclerosis, thrombosis, and endothelial dysfunction (James and Bennett, 2019). Our research also supports that Men are more affected by these mechanisms. But some researchers (Dai et al., 2022) suggest that men’s greater tendency to smoke excessively may also contribute to this. This standpoint also is consistent with our study’s findings, which indicate that women in high-income regions are disproportionately affected by smoking-related health impacts across three regions. According to a survey report on smoking, female consumption remains highest in the high SDI group, approximately twice the level observed in other SDI categories in 2020, although consumption has decreased by around 60% since 1970 (Dai et al., 2022).

Meanwhile, metabolic risks are the primary risk factor for women across all age groups, becoming more pronounced with age. The association between sex differences and cardiovascular risk factors in AD suggests that although conditions like hypertension, hypercholesterolemia, and diabetes increase the risk of cardiovascular disease and AD in both sexes, women with these risk factors appear to face a higher risk of developing AD compared to men (Deckers et al., 2015). This is linked to the loss of estrogen’s protective effects in older women. These findings underscore the importance of developing gender-specific dementia prevention and management strategies, particularly those that account for changes in risk factors following menopause.

While the Global Burden of Disease (GBD) database currently lacks systematic documentation on the association between alcohol consumption and ADRD, emerging evidence identifies alcohol intake as a modifiable risk factor for ADRD. A population-based longitudinal cohort study found that both chronic high-dose alcohol consumption (≥30 units per week; 1 unit = 8 g ethanol) and moderate drinking (14–21 units per week) were associated with accelerated hippocampal volume loss compared with light or non-drinkers (Topiwala et al., 2017). Although some studies suggest that light to moderate alcohol intake may have protective effects against ADRD in certain populations (Laughlin et al., 2016), traditional drinking patterns in East and Southeast Asia often involve high-alcohol-content spirits, which may contribute to the elevated disease burden in these regions.

Although dementia risk results from the complex interplay of age and genetic factors—both uncontrollable—modifiable risk factors like low educational attainment, hypertension, type 2 diabetes, alcohol consumption, smoking, and nutritional deficiencies significantly impact the YLD of Alzheimer’s disease and other dementias (Yu-Tzu et al., 2016; Zhou and Wang, 2021). Despite the rise in population aging and chronic diseases, a 2016 study in high-income Western European countries reported that dementia prevalence had stabilized or declined in the past 2 years, attributed to improvements in education, living conditions, lifestyle, and reductions in chronic diseases (Tini et al., 2020). These findings align with our research on HIAP regions, where the YLD of dementia has similarly shown a trend toward stabilization or decline as the SDI has advanced, reinforcing our confidence in lifestyle interventions to prevent and manage dementia.

In addition to avoiding negative factors, many positive measures should be advocated. A study on multi-domain interventions for preventing cognitive decline has shown that multi-domain interventions combining exercise, diet, cognitive training, and cardiovascular management can effectively improve or maintain cognitive function in high-risk older adults within the general population (Ngandu et al., 2015). The Mediterranean diet has been proven to play a positive role in preventing the development of dementia, while a specialized neuroprotective dietary plan has been shown to significantly slow the age-related decline in cognitive abilities (Tari et al., 2025). A recent authoritative review systematically discussed how regular exercise mediates neuroprotective effects through mechanisms such as improving brain blood flow, reducing inflammation, and enhancing neuroplasticity, particularly in dementia protection. It strongly recommends endurance exercises that promote cardiovascular health as a key measure to prevent and slow age-related cognitive decline (Tari et al., 2025). Some skill-intensive activities, such as social dancing and Tai Chi, are especially encouraged, as they stimulate both physical exercise and brain learning and thinking, enhancing brain flexibility (Coubard, 2011). Additionally, factors such as increasing social interactions (Holwerda et al., 2014), maintaining good sleep habits (Xie et al., 2013), and exposure to healthy living environments and air quality are also considered significant in preventing cognitive decline (Huang et al., 2025).

In recent years, the global and regional YLD of dementia in East Asia and high-income regions has stabilized or slightly declined with the development of the SDI. However, the relationship between dementia YLD and SDI in China is unique, as the disease burden has significantly increased alongside SDI development, without showing signs of slowing down. This may require further advanced analyses and predictive studies. High-income Asian countries such as Japan and South Korea also experienced an initial abnormal increase in YLD with rising SDI, followed by stabilization and decline. However, the exceptionally high YLD in China, coupled with SDI changes, highlights the need for greater attention and focus on addressing this issue.

Notably, some countries in East Asia and Southeast Asia, such as China, Japan, and South Korea, have already developed their national dementia plans based on their specific conditions and the latest research progress. These plans aim to increase public awareness of Alzheimer’s disease and promote early screening and intervention (Hampel et al., 2022). However, further epidemiological and interdisciplinary research is required to comprehensively evaluate and refine these policies, with a targeted focus on gender-specific disparities, to ensure their contextual efficacy across diverse national settings. In particular, China has a long history of mind-stimulating practices—such as Xiangqi (Chinese chess), Go, abacus calculation, and calligraphy—that have been cultivated over centuries to develop logical reasoning, memory, and executive function from childhood through adolescence. These culturally rooted methods warrant greater emphasis in both policy formulation and future research (Yongxia, 2023). Furthermore, the value of neuroimaging in early diagnosis and intervention should be more strongly emphasized in public health policy. Functional MRI (fMRI) and diffusion tensor imaging (DTI) studies have demonstrated reduced connectivity from both the posterior cingulate cortex (PCC) and the hippocampus to the rest of the brain in individuals with mild cognitive impairment (MCI) and Alzheimer’s disease (AD), as well as decreased connectivity between these two regions. This pattern may serve as a promising early imaging biomarker for AD, emerging earlier than many conventional diagnostic indicators (Zhou et al., 2008).

Our findings provide a detailed understanding of the gender and regional dimensions of AD burden, offering actionable insights for policymakers. By identifying the interplay of socio-demographic, hormonal, and lifestyle factors, this study emphasizes the potential for targeted interventions to mitigate the growing dementia threat in NHIEA and NHISEA. However, several limitations cannot be overlooked. First, the data used here do not clearly distinguish between several unique forms of dementia, which may affect targeted management and control in real-world applications. Second, although the 2021 GBD study strictly follows the ICD-10 guidelines, misdiagnosis of Alzheimer’s disease and other dementias cannot be entirely ruled out due to limited medical resources and patient non-compliance in local clinical practices. The relative subjectivity of dementia symptom diagnoses across countries and regions may also introduce discrepancies. Finally, our current work focuses only on regional and national levels in NHIEA and NHISEA, meaning the results may not fully apply to populations living on other continents. Further comparative studies between continents are needed. Although a study on Years Lived with Disability Trend Analysis in Asian and North African countries (Amini et al., 2019) has been conducted, there is still a gap in analyzing gender differences in Alzheimer’s disease and other dementias across continents, a disease with notable gender disparities.

## 5 Conclusion

The health burden of Alzheimer’s disease and other dementias remains significant, with distinct patterns observed across NHIEA, NHISEA, and HIAP, which include regional variations in gender, age, and risk factors. These findings highlight the need for tailored approaches to allocate healthcare resources and implement appropriate control measures based on each region’s specific conditions. Greater emphasis should be placed on managing modifiable risk factors, such as smoking, high body mass index, and elevated fasting blood glucose, through targeted public health initiatives. In particular, age and gender differences—especially those related to menopause in women—should be prioritized, as these factors can affect the effectiveness of interventions. Future research should continue exploring the regional differences in disease burden, particularly gender disparities, to refine prevention and treatment strategies and further optimize healthcare policies.

## Data Availability

The original contributions presented in the study are included in the article/Supplementary material, further inquiries can be directed to the corresponding authors.

## Footnotes

^1^ http://ghdx.healthdata.org/GBD-results-tool
