# Supplementary material for: Age-sex differences in Alzheimer’s and related dementias burden and risk factors in east and Southeast Asia: results from the 2021 GBD study
Source: Front Aging Neurosci. 2025 Jun 27;17:1562148. doi: 10.3389/fnagi.2025.1562148 (PMC12245908; doi:10.3389/fnagi.2025.1562148)
Supplement: Supplementary file 1 [file Data_Sheet_1.pdf]

Catalogue

Table S1. The age standardized prevalence rates and percentage changes of Alzheimer's disease and other dementias by gender in East and Southeast Asia, 1990 and 2021
Table S2. The age standardized incidence rates and incidence changes of Alzheimer's disease and other dementias by gender in East and Southeast Asia, 1990 and 2021
Table S3. The age standardized YLDs rates and YLDs changes of Alzheimer's disease and other dementias by gender in East and Southeast Asia, 1990 and 2021
Table S4.The absolute numbers and rates of prevalence of Alzheimer's disease and other dementiase in different age groups in Non-High-income East Asia in 2021
Table S5.The absolute numbers and rates of prevalence of Alzheimer's disease and other dementiase in different age groups in Non-High-income Southeast Asia in 2021
Table S6.The absolute numbers and rates of prevalence of Alzheimer's disease and other dementiase in different age groups in High-income Asia Pacific in 2021
Table S7.The absolute numbers and rates of incidence of Alzheimer's disease and other dementiase in different age groups in Non-High-income East Asia in 2021
Table S8.The absolute numbers and rates of incidence of Alzheimer's disease and other dementiase in different age groups in Non-High-income Southeast Asia in 2021
Table S9.The absolute numbers and rates of incidence of Alzheimer's disease and other dementiase in different age groups in High-income Asia Pacific in 2021
Table S10.The absolute numbers and rates of YLDs of Alzheimer's disease and other dementiase in different age groups in Non-High-income East Asia in 2021
Table S11.The absolute numbers and rates of YLDs of Alzheimer's disease and other dementiase in different age groups in Non-High-income Southeast Asia in 2021
Table S12.The absolute numbers and rates of YLDs of Alzheimer's disease and other dementiase in different age groups in High-income Asia Pacific in 2021
Table S13.The ratio of male to female prevalence, incidence, and YLDs rates of Alzheimer's disease and other dementiase according to different age groups in Non-High-income East Asia
Table S14.The ratio of male to female prevalence, incidence, and YLDs rates of Alzheimer's disease and other dementiase according to different age groups in Non-High-income Southeast Asia
Table S15.The ratio of male to female prevalence, incidence, and YLDs rates of Alzheimer's disease and other dementiase according to different age groups in High-income Asia Pacific
Table S16.The SDI and age-standardized YLD rates for Both with Alzheimer's disease and other dementias from 1990 to 2019.
Table S17.The SDI and age-standardized YLD rates for Male with Alzheimer's disease and other dementias from 1990 to 2019.

Table S18.The SDI and age-standardized YLD rates for Female with Alzheimer's disease and other dementias from 1990 to 2019.
Table S19. Risk Factors for Alzheimer’ s Disease and Other Dementias in Both Sexes in Non-High-income East Asia , 2021
Table S20. Risk Factors for Alzheimer’ s Disease and Other Dementias in Male Sexes in Non-High-income East Asia , 2021
Table S21. Risk Factors for Alzheimer’ s Disease and Other Dementias in Female Sexes in Non-High-income East Asia , 2021
Table S22. Risk Factors for Alzheimer’ s Disease and Other Dementias in Both Sexes in Non-High-income Southeast Asia , 2021
Table S23. Risk Factors for Alzheimer’ s Disease and Other Dementias in Male Sexes in Non-High-income Southeast Asia , 2021
Table S24. Risk Factors for Alzheimer’ s Disease and Other Dementias in Female Sexes in Non-High-income Southeast Asia , 2021
Table S25. Risk Factors for Alzheimer’ s Disease and Other Dementias in Both Sexes in High-income Asia Pacific , 2021
Table S26. Risk Factors for Alzheimer’ s Disease and Other Dementias in Male Sexes in High-income Asia Pacific , 2021
Table S27. Risk Factors for Alzheimer’ s Disease and Other Dementias in Female Sexes in High-income Asia Pacific , 2021

Table S1. The age standardized prevalence rates and percentage changes of Alzheimer's disease and other dementias by gender in East and Southeast Asia, 1990 and 2021

Country/Region	Male			Female		
	1990	2021	Change rate	1990	2021	Change rate
Non-High-income East Asia	568.91 (488.79,659.03)	719.43 (608.42,838.07)	26.46%(21.55%,29.81%)	779.02 (675.2,893.04)	1010.3 (866.63,1168.43)	29.69%(25.78%,32.94%)
People's Republic of China	574.55 (493.64,666.55)	731.21 (618.54,851.63)	27.27%(22.21%,30.75%)	785.19 (681.22,900.41)	1025.11 (879.04,1186.81)	30.56%(26.49%,33.89%)
Democratic People's Republic of Korea	488.84 (412.84,564.77)	480.31 (408.47,552.84)	-1.75%(-6.3%,2.95%)	690.03 (582.58,792.21)	675.18 (577.1,777.41)	-2.15%(-6.92%,2.41%)
Taiwan (Province of China)	411.08 (350.28,472.18)	427.33 (357.33,483.25)	3.95%(-3.19%,11.86%)	590.34 (509.22,676.02)	654.96 (559.78,737.65)	10.95%(0.75%,24.19%)
Non-High-income Southeast Asia	578.96 (502.81,659.67)	544.76 (469.62,624.89)	-5.91%(-7.6%,-4.49%)	745.57 (650.53,850.71)	714.77 (621.33,819.49)	-4.13%(-5.64%,-2.54%)
Kingdom of Cambodia	585.54 (505.79,672.26)	551.97 (472.53,631.52)	-5.73%(-10.06%,-1.27%)	744.37 (639.53,848.89)	717.59 (618.08,823.96)	-3.6%(-7.73%,1.25%)
Republic of Indonesia	592.24 (511.99,680.41)	568.34 (491.41,654)	-4.04%(-5.85%,-2.21%)	749.23 (646.89,860.31)	733.81 (632.66,843.57)	-2.06%(-3.84%,-0.16%)
Lao People's Democratic Republic	579.35 (497.71,665.95)	562.05 (481.51,645.34)	-2.99%(-7.38%,1.86%)	738.19 (634.58,846.37)	728.38 (629.57,835.37)	-1.33%(-5.8%,3.8%)
Malaysia	569.2 (481.18,653.09)	547.52 (467.26,629.44)	-3.81%(-8.52%,0.35%)	806 (695.85,917.7)	767.29 (654.89,883.38)	-4.8%(-9.35%,-0.05%)
Republic of Maldives	592.63 (505.71,681.04)	579.6 (488.46,667.44)	-2.2%(-7.07%,2.28%)	760.57 (655.7,871.75)	763.15 (658.22,873.33)	0.34%(-4.26%,5.55%)
Republic of the Union of Myanmar	607.42 (522.81,696.91)	547.79 (467.31,631.97)	-9.82%(-14.15%,-5.46%)	802.93 (696.44,914.58)	735.91 (632.41,843.3)	-8.35%(-12.37%,-4.06%)
Republic of the Philippines	606.97 (528,699.02)	562.68 (488.02,648.66)	-7.3%(-8.45%,-6.34%)	780.73 (677.91,889.61)	732.91 (636.57,842.22)	-6.13%(-7.09%,-5.17%)
Democratic Socialist Republic of Sri Lanka	601.13 (511.02,688.45)	560.36 (473.77,651.27)	-6.78%(-11.68%,-2.6%)	717.8 (622.33,821.02)	682.35 (583.73,783.91)	-4.94%(-9.4%,-0.07%)
Kingdom of Thailand	505.64 (433.34,578.67)	507.54 (428.89,585.73)	0.38%(-4.59%,5.51%)	667.91 (581.81,754)	674.86 (582.87,775.11)	1.04%(-4.27%,6.74%)
Democratic Republic of Timor-Leste	623.76 (533.39,712.89)	577.12 (490.15,661.98)	-7.48%(-11.75%,-2.73%)	793.18 (683.44,903.46)	747.67 (638.92,848.45)	-5.74%(-10.25%,-0.95%)
Socialist Republic of Viet Nam	572.64 (490.33,657.33)	532.48 (454.35,618.34)	-7.01%(-12.78%,-2.29%)	751.72 (646.96,860.02)	714.14 (621.05,814.1)	-5%(-9.23%,-0.87%)
Mauritius	558.12 (472.89,647.17)	551.56 (470.26,635.95)	-1.18%(-5.86%,3.71%)	721.67 (619.37,827.4)	727.87 (626.29,829.46)	0.86%(-3.95%,5.77%)
Seychelles	551.04 (464.74,640.91)	535.5 (453.73,625.44)	-2.82%(-7.35%,1.67%)	735.94 (632.86,844.19)	715.92 (609.9,822.04)	-2.72%(-7.01%,1.91%)
High-income Asia Pacific	540.48 (466.84,615.87)	564.24 (490.73,646.6)	4.4%(1.91%,6.94%)	727.64 (634.58,825.09)	767.81 (670.03,872.59)	5.52%(3.26%,7.78%)
Brunei Darussalam	513.77 (439.85,593.24)	504.63 (429.99,577.67)	-1.78%(-6.49%,3.08%)	634.73 (538.01,732.85)	627.8 (541.2,723.54)	-1.09%(-5.9%,4.66%)
Japan	536.39 (463.45,613.48)	551.02 (477.19,631.94)	2.73%(0.4%,5.19%)	718.87 (625.93,817.62)	759.99 (657.37,866.96)	5.72%(3.66%,7.74%)
Republic of Korea	592.39 (517.08,665.62)	638.88 (553.57,728.84)	7.85%(0.58%,14.51%)	816.39 (706.59,930.5)	805.1 (697.64,907.35)	-1.38%(-7.62%,5.95%)
Republic of Singapore	452.61 (392.99,515.13)	470.44 (419.16,527.5)	3.94%(-2.42%,10.2%)	557.72 (478.55,629.29)	582.19 (517.62,641.64)	4.39%(-1.54%,12.67%)

Table S2. The age standardized incidence rates and incidence changes of Alzheimer's disease and other dementias by gender in East and Southeast Asia, 1990 and 2021						
**Country/Region**	**Male**			**Female**		
	**1990**	**2021**	**Change rate**	**1990**	**2021**	**Change rate**
Non-High-income East Asia	99.42 (85.85,114.23)	124.72 (106.27,143.6)	25.45%(20.98%,28.71%)	134.55 (117.62,153.1)	169.64 (148.11,193.44)	26.08%(22.53%,29.21%)
People's Republic of China	100.25 (86.5,115.16)	126.48 (107.78,145.62)	26.17%(21.63%,29.56%)	135.42 (118.39,154.07)	171.81 (150.12,195.9)	26.87%(23.21%,29.95%)
Democratic People's Republic of Korea	87.11 (73.81,100.58)	85.7 (73.49,99.12)	-1.62%(-6.48%,3.03%)	121.05 (104.5,139.48)	118.67 (102.56,136.84)	-1.97%(-6.65%,2.26%)
Taiwan (Province of China)	74.88 (64.08,85.87)	76.4 (64.75,86.64)	2.03%(-4.95%,10.53%)	105.86 (91.45,121.13)	115.21 (98.76,130.11)	8.83%(-0.69%,21.43%)
Non-High-income Southeast Asia	99.3 (86.65,113.52)	93.82 (81.63,107.74)	-5.51%(-7.12%,-3.94%)	126.59 (111.36,144.01)	121.87 (107.02,139)	-3.73%(-5.02%,-2.39%)
Kingdom of Cambodia	99.97 (86.97,114.92)	94.57 (82.07,108.64)	-5.4%(-9.6%,-0.97%)	126.42 (110.36,143.83)	122.3 (106.21,139.93)	-3.26%(-7.39%,0.9%)
Republic of Indonesia	102.02 (88.79,116.18)	98.3 (85.03,112.13)	-3.64%(-5.25%,-2.07%)	127.53 (111.11,145.57)	125.18 (108.78,143.22)	-1.84%(-3.33%,-0.42%)
Lao People's Democratic Republic	98.89 (86.33,114.37)	96.34 (83.02,111.07)	-2.57%(-6.73%,2.05%)	125.34 (110.27,143.17)	124.19 (108.08,141.97)	-0.92%(-5.16%,3.56%)
Malaysia	96.42 (82.8,110.3)	93.6 (79.8,107.89)	-2.93%(-7.22%,1.73%)	133.57 (116.99,152.05)	128.69 (111.93,146.91)	-3.65%(-7.51%,0.43%)
Republic of Maldives	100.38 (86.92,115.8)	98.6 (83.99,114.42)	-1.77%(-6.38%,2.64%)	128.21 (111.84,146.16)	129.26 (113.46,148.24)	0.82%(-3.43%,5.92%)
Republic of the Union of Myanmar	103.82 (90.78,119.41)	93.98 (80.74,108.41)	-9.48%(-13.59%,-5.19%)	136.06 (119.54,154.25)	125.41 (110.41,142.24)	-7.83%(-11.27%,-4.08%)
Republic of the Philippines	104.58 (91.22,119.36)	97.26 (84.22,111.41)	-7%(-8.1%,-6.11%)	133.44 (117.63,151.39)	125.61 (110.48,142.58)	-5.87%(-6.67%,-5.13%)
Democratic Socialist Republic of Sri Lanka	101.41 (87.97,116.51)	94.99 (81.52,109.82)	-6.34%(-10.7%,-2.11%)	121.37 (106.2,138.58)	115.87 (101.08,132.55)	-4.53%(-8.5%,-0.12%)
Kingdom of Thailand	86.87 (75.01,99.16)	87.44 (75.42,101.14)	0.65%(-4.04%,5.53%)	113.7 (99.81,129.19)	115.35 (101.02,131.53)	1.45%(-3.33%,6.58%)
Democratic Republic of Timor-Leste	106.05 (92.33,121.94)	98.68 (85.69,114.7)	-6.95%(-11.34%,-2.51%)	134.1 (116.79,152.11)	127.04 (110.08,145.84)	-5.27%(-9.18%,-0.98%)
Socialist Republic of Viet Nam	97.88 (84.47,112.49)	91.3 (78.32,106.46)	-6.72%(-11.61%,-1.77%)	127.03 (111.11,145.19)	121.03 (105.92,138.19)	-4.72%(-8.48%,-0.82%)
Mauritius	95.39 (81.9,111.02)	94.56 (80.93,109.02)	-0.87%(-5.11%,3.84%)	122.36 (106.56,141.17)	123.63 (107.29,141.19)	1.04%(-3.76%,5.94%)
Seychelles	94.33 (80.46,109.87)	91.94 (77.54,107.76)	-2.53%(-7.13%,1.75%)	124.61 (108.44,141.86)	121.5 (106.08,139.92)	-2.49%(-6.92%,2%)
High-income Asia Pacific	97.55 (84.76,111.5)	98.53 (85.85,112.48)	1%(-1.1%,3.21%)	128.77 (113.13,146.35)	133.05 (116.34,151.45)	3.32%(1.3%,5.45%)
Brunei Darussalam	90.42 (78.7,105.24)	88.76 (76.59,102.1)	-1.83%(-6.06%,3%)	111.84 (96.03,129.27)	110.63 (96.01,128.08)	-1.08%(-5.69%,3.91%)
Japan	97.05 (84.09,110.89)	96.4 (83.61,110.23)	-0.67%(-2.76%,1.22%)	127.58 (111.45,145.28)	132.39 (115.67,151)	3.77%(1.98%,5.55%)
Republic of Korea	102.76 (90.58,116.58)	108.86 (95.78,123.87)	5.94%(-0.23%,12.38%)	139.19 (121.97,158.47)	135.21 (117.94,152.7)	-2.86%(-9.07%,4.41%)
Republic of Singapore	80.82 (70.99,90.66)	83.72 (74.48,94.04)	3.59%(-1.97%,9.32%)	98.85 (86.22,111.19)	103.48 (93.28,113.88)	4.69%(-1.76%,12.43%)

Table S3. The age standardized YLDs rates and YLDs changes of Alzheimer's disease and other dementias by gender in East and Southeast Asia, 1990 and 2021						
**Country/Region**	**Male**			**Female**		
	**1990**	**2021**	**Change rate**	**1990**	**2021**	**Change rate**
Non-High-income East Asia	112.07 (76.73,147.77)	141.31 (97.94,189.04)	26.09%(21.21%,29.56%)	165.35 (113.06,220.52)	213.32 (145.77,284.69)	29.01%(25.21%,32.12%)
People's Republic of China	113.18 (77.45,149.5)	143.7 (99.59,192.13)	26.96%(21.93%,30.56%)	166.61 (114.06,222.28)	216.38 (147.99,288.88)	29.87%(25.94%,33.04%)
Democratic People's Republic of Korea	97.44 (66.35,131.49)	95.67 (65.3,127)	-1.82%(-7.72%,4.7%)	148.08 (101.41,195.21)	145.1 (99.42,193.39)	-2.01%(-7.63%,3.78%)
Taiwan (Province of China)	81.3 (55.82,108.62)	84.12 (58.26,111.09)	3.47%(-5.63%,12.9%)	126.08 (86.06,168)	139.62 (98.86,186.79)	10.74%(0.06%,25.13%)
Non-High-income Southeast Asia	111.86 (77.47,146.81)	105.45 (72.76,139.85)	-5.73%(-7.91%,-3.82%)	155.98 (107,207.83)	149.69 (102.12,198)	-4.03%(-5.87%,-2.03%)
Kingdom of Cambodia	112.44 (78.02,148.89)	106.27 (73.23,140.48)	-5.49%(-11.65%,0.77%)	154.32 (106.4,204.26)	149.18 (102.13,199.67)	-3.33%(-9.3%,2.82%)
Republic of Indonesia	114.48 (79.14,150.63)	110.23 (76.6,145.76)	-3.71%(-6.01%,-1.76%)	156.42 (108.83,208.12)	153.52 (105.07,204.03)	-1.86%(-4.06%,0.36%)
Lao People's Democratic Republic	113.26 (78.38,150.05)	110.03 (75.89,146.7)	-2.85%(-8.57%,3.9%)	156.33 (106.85,207.93)	153.94 (106.19,204.65)	-1.53%(-6.78%,4.83%)
Malaysia	109.91 (75.56,144.96)	105.36 (72.32,140.67)	-4.14%(-10.53%,1.71%)	168.06 (115.87,222.27)	159.42 (110.2,210.31)	-5.14%(-10.63%,0.41%)
Republic of Maldives	115.62 (79.65,154.25)	113.1 (77.46,151.08)	-2.18%(-8.23%,4.03%)	159.89 (109.85,212.4)	160.71 (110.68,210.38)	0.51%(-4.95%,6.65%)
Republic of the Union of Myanmar	116.51 (80.81,151.72)	105.49 (72.68,138.85)	-9.46%(-15.1%,-3.71%)	167.48 (115.7,223.59)	153.08 (105.83,202.01)	-8.6%(-13.7%,-2.74%)
Republic of the Philippines	116.38 (80.58,153.31)	109.01 (75.23,144.15)	-6.33%(-7.61%,-5%)	161.91 (112.05,215.19)	152.82 (105.14,202.85)	-5.61%(-6.95%,-4.48%)
Democratic Socialist Republic of Sri Lanka	116.07 (80.97,154.07)	106.67 (72.75,142.92)	-8.09%(-13.8%,-2.74%)	150.04 (103.74,197.29)	140.87 (96.46,184.46)	-6.11%(-11.92%,0.47%)
Kingdom of Thailand	97.62 (67.03,128.81)	98.4 (67.16,129.39)	0.81%(-6.12%,7.97%)	140.33 (96.98,187.95)	142.29 (97.93,188.72)	1.4%(-5.39%,7.63%)
Democratic Republic of Timor-Leste	119.73 (82.59,157.61)	110.32 (76.84,145.62)	-7.85%(-13.75%,-1.86%)	164.69 (112.56,218)	155.59 (108.32,205.01)	-5.53%(-10.94%,0.83%)
Socialist Republic of Viet Nam	111.9 (77.08,147.36)	104.11 (71.18,140.86)	-6.96%(-13.76%,-0.71%)	158.42 (108.83,208.71)	150.7 (103.2,201.14)	-4.87%(-9.76%,0.24%)
Mauritius	108.35 (73.99,143.83)	105.53 (73.06,140.21)	-2.6%(-8.63%,4.33%)	151.3 (104.38,203.04)	150.74 (103.47,196.41)	-0.37%(-6.55%,5.51%)
Seychelles	107.53 (73.85,145.2)	102.43 (71.19,138.27)	-4.74%(-11.01%,0.82%)	156.27 (106.82,208.67)	148.74 (101.97,195.53)	-4.82%(-10.04%,1.03%)
High-income Asia Pacific	105.48 (72.27,139.79)	110.53 (75.74,147.21)	4.79%(2.16%,7.6%)	156.16 (106.27,207.62)	165.5 (112.42,220.67)	5.98%(3.52%,8.54%)
Brunei Darussalam	99.14 (69.43,133.11)	96.9 (66.69,128.52)	-2.26%(-7.69%,4.43%)	135.1 (92.73,181.31)	132.98 (90.82,177.17)	-1.57%(-7.45%,5%)
Japan	104.87 (71.87,139.12)	108.18 (74.13,144.41)	3.16%(0.45%,6.03%)	154.5 (105.25,204.99)	164.29 (112.59,219.88)	6.34%(4.13%,8.57%)
Republic of Korea	113.72 (79.24,149.58)	124.05 (85.27,163.34)	9.09%(1.26%,17.48%)	173.48 (119.08,230.88)	171.19 (117.32,226.14)	-1.32%(-8.75%,6.81%)
Republic of Singapore	89.11 (61.58,117.25)	93.58 (64.74,121.28)	5.02%(-2.01%,13.24%)	120.73 (83.4,161.97)	126.86 (89.29,167.4)	5.07%(-1.86%,14.05%)

Table S4.The absolute numbers and rates of prevalence of Alzheimer's disease and other dementiase in different age groups in Non-High-income East Asia in 2021				
**Age**	**Male (Numbers, 95% UI)**	**Female (Numbers, 95% UI)**	**Male (Rates, 95% UI)**	**Female Rates, 95% UI)**
40-44 years	8581 (3535,15100)	9451 (3809,15949)	17.55 (7.23,30.88)	20.32 (8.19,34.3)
45-49 years	68858 (43198,104120)	77120 (48711,114878)	118.86 (74.57,179.73)	137.4 (86.79,204.67)
50-54 years	197674 (137692,267233)	224771 (160347,302809)	313.35 (218.27,423.61)	364.21 (259.82,490.66)
55-59 years	377023 (289773,482877)	439523 (344270,570321)	666.46 (512.23,853.58)	773.14 (605.59,1003.22)
60-64 years	489987 (372829,624769)	573930 (440650,734220)	1287.62 (979.75,1641.81)	1511.96 (1160.85,1934.22)
65-69 years	844346 (626356,1070025)	1123839 (863939,1408568)	2173.77 (1612.55,2754.78)	2791.07 (2145.6,3498.2)
70-74 years	961751 (704873,1262655)	1506845 (1155197,1938254)	3617.27 (2651.12,4749.01)	5308.17 (4069.42,6827.9)
75-79 years	1126730 (880176,1401574)	1954784 (1544466,2408533)	7024.53 (5487.41,8738.03)	10722.28 (8471.63,13211.17)
80-84 years	1183033 (923438,1495250)	2287110 (1801672,2860913)	13192.29 (10297.47,16673.89)	19645.51 (15475.76,24574.29)
85-89 years	754362 (588479,945796)	1846381 (1452614,2288002)	20894.16 (16299.55,26196.46)	29238.58 (23003.04,36231.91)
90-94 years	235282 (183263,298510)	833098 (661276,1032090)	27045.33 (21065.88,34313.3)	37425.35 (29706.55,46364.66)
95+ years	44082 (32930,55696)	245611 (189717,305321)	29552.84 (22076.56,37339.18)	44355.33 (34261.38,55138.45)

Table S5.The absolute numbers and rates of prevalence of Alzheimer's disease and other dementiase in different age groups in Non-High-income Southeast Asia in 2021				
**Age**	**Male (Numbers, 95% UI)**	**Female (Numbers, 95% UI)**	**Male (Rates, 95% UI)**	**Female Rates, 95% UI)**
40-44 years	4422 (1950,7487)	4938 (2117,8379)	17.95 (7.91,30.38)	20.22 (8.67,34.3)
45-49 years	25973 (16390,38676)	29943 (18925,44310)	117.42 (74.1,174.85)	134.34 (84.91,198.8)
50-54 years	59088 (41725,81063)	71114 (51776,96505)	297.58 (210.13,408.24)	347.98 (253.35,472.22)
55-59 years	99712 (77108,127698)	126029 (98798,161804)	589.16 (455.6,754.52)	703.36 (551.39,903.03)
60-64 years	143454 (110715,181528)	191044 (148306,240626)	1050.74 (810.94,1329.62)	1282.08 (995.26,1614.81)
65-69 years	170535 (131536,216750)	247109 (193091,310968)	1735.39 (1338.53,2205.68)	2195.8 (1715.8,2763.25)
70-74 years	179445 (136559,233005)	287617 (223108,365736)	2915.9 (2219.02,3786.22)	3873.13 (3004.44,4925.11)
75-79 years	187350 (146391,230997)	343612 (268862,422307)	5312.95 (4151.42,6550.72)	7252.59 (5674.85,8913.59)
80-84 years	177495 (138586,224114)	371425 (292470,467854)	9318.2 (7275.54,11765.63)	12712.47 (10010.14,16012.87)
85-89 years	123308 (95715,155324)	282264 (219029,352248)	14400.54 (11178.13,18139.57)	19150.04 (14859.94,23898.07)
90-94 years	57920 (45278,73072)	136897 (107723,171297)	19374.79 (15146.04,24443.25)	25394.34 (19982.61,31775.67)
95+ years	20920 (15618,26625)	46185 (35503,58878)	23489.1 (17535.14,29894.13)	30830.3 (23699.88,39303.54)

Table S6.The absolute numbers and rates of prevalence of Alzheimer's disease and other dementiase in different age groups in High-income Asia Pacific in 2021				
**Age**	**Male (Numbers, 95% UI)**	**Female (Numbers, 95% UI)**	**Male (Rates, 95% UI)**	**Female Rates, 95% UI)**
40-44 years	1039 (441,1803)	1045 (395,1842)	15.53 (6.6,26.96)	16.37 (6.19,28.85)
45-49 years	8035 (5001,12080)	9200 (5398,13647)	108.1 (67.28,162.52)	128.26 (75.26,190.26)
50-54 years	18909 (13039,25886)	23644 (16764,32721)	262.17 (180.78,358.89)	333.72 (236.62,461.85)
55-59 years	31851 (24512,40711)	40611 (31708,51431)	508.76 (391.53,650.27)	653.09 (509.91,827.1)
60-64 years	55717 (43226,70401)	73590 (57132,92494)	947.62 (735.17,1197.36)	1225.12 (951.12,1539.83)
65-69 years	92108 (71381,115726)	125412 (99613,156382)	1690.85 (1310.36,2124.41)	2180.54 (1731.98,2719.01)
70-74 years	177859 (138617,227829)	255471 (201787,323542)	3112.25 (2425.58,3986.63)	4014.26 (3170.71,5083.87)
75-79 years	224809 (178278,276994)	371784 (295972,453209)	5955.62 (4722.94,7338.1)	7948.34 (6327.56,9689.11)
80-84 years	288275 (228382,359685)	573666 (457611,715862)	10331.49 (8184.99,12890.73)	14416.34 (11499.88,17989.76)
85-89 years	243566 (188919,305781)	630537 (495570,782217)	15163.41 (11761.32,19036.69)	21787.4 (17123.79,27028.5)
90-94 years	125317 (97720,160175)	440746 (345112,555916)	19407.89 (15134.06,24806.4)	28430.48 (22261.6,35859.53)
95+ years	55885 (41982,71689)	240449 (183903,308630)	23465.66 (17627.93,30101.75)	34151.97 (26120.58,43836.09)

Table S7.The absolute numbers and rates of incidence of Alzheimer's disease and other dementiase in different age groups in Non-High-income East Asia in 2021				
**Age**	**Male (Numbers, 95% UI)**	**Female (Numbers, 95% UI)**	**Male (Rates, 95% UI)**	**Female Rates, 95% UI)**
40-44 years	5097 (2101,8973)	5619 (2266,9479)	10.42(4.3,18.35)	12.08(4.87,20.38)
45-49 years	18153 (11169,27220)	20459 (12656,30742)	31.34(19.28,46.99)	36.45(22.55,54.77)
50-54 years	38547 (24784,54993)	43866 (28213,61851)	61.1(39.29,87.17)	71.08(45.72,100.22)
55-59 years	70809 (46791,99208)	81400 (53335,115052)	125.17(82.71,175.37)	143.19(93.82,202.38)
60-64 years	80668 (54888,110150)	98647 (65710,137630)	211.99(144.24,289.46)	259.87(173.11,362.57)
65-69 years	126232 (83677,182813)	199300 (134922,285885)	324.98(215.43,470.65)	494.96(335.08,710)
70-74 years	160463 (106257,225635)	281780 (193394,389187)	603.52(399.65,848.64)	992.63(681.27,1370.99)
75-79 years	213723 (142184,293450)	372702 (257428,508722)	1332.44(886.44,1829.49)	2044.32(1412.03,2790.42)
80-84 years	219119 (150138,292665)	379924 (265517,503083)	2443.45(1674.22,3263.57)	3263.42(2280.71,4321.32)
85-89 years	124786 (88053,165784)	261728 (186066,346881)	3456.3(2438.86,4591.86)	4144.63(2946.47,5493.07)
90-94 years	36030 (25396,49419)	111610 (79625,153966)	4141.59(2919.25,5680.63)	5013.88(3577.01,6916.64)
95+ years	6687 (4099,9730)	31377 (19670,45790)	4483.01(2748.25,6522.85)	5666.39(3552.25,8269.34)

Table S8.The absolute numbers and rates of incidence of Alzheimer's disease and other dementiase in different age groups in Non-High-income Southeast Asia in 2021				
**Age**	**Male (Numbers, 95% UI)**	**Female (Numbers, 95% UI)**	**Male (Rates, 95% UI)**	**Female (Rates, 95% UI)**
40-44 years	2641 (1163,4466)	2950 (1264,4999)	10.72(4.72,18.13)	12.08(5.18,20.46)
45-49 years	6871 (4262,10268)	8004 (5087,11853)	31.06(19.27,46.42)	35.91(22.82,53.18)
50-54 years	11081 (7132,15655)	13503 (8851,18904)	55.8(35.92,78.84)	66.07(43.31,92.5)
55-59 years	16687 (11018,23448)	21283 (14049,29476)	98.6(65.1,138.55)	118.78(78.41,164.51)
60-64 years	21602 (14433,29840)	29504 (19690,40937)	158.22(105.72,218.57)	198(132.14,274.73)
65-69 years	25443 (16574,36685)	39030 (25689,55529)	258.91(168.66,373.31)	346.82(228.27,493.43)
70-74 years	28528 (19311,39380)	48772 (33655,67191)	463.57(313.79,639.91)	656.78(453.21,904.81)
75-79 years	32235 (21220,44583)	60590 (41591,83384)	914.14(601.76,1264.3)	1278.86(877.86,1759.98)
80-84 years	31885 (21334,43234)	64146 (43591,86538)	1673.89(1119.98,2269.7)	2195.49(1491.94,2961.86)
85-89 years	21923 (15160,29873)	46754 (32866,62390)	2560.25(1770.46,3488.79)	3171.97(2229.76,4232.83)
90-94 years	10387 (7364,14245)	22903 (16218,31533)	3474.58(2463.35,4764.99)	4248.55(3008.39,5849.35)
95+ years	3801 (2377,5426)	7720 (4945,11062)	4268.23(2668.93,6091.83)	5153.46(3301.26,7384.23)

Table S9.The absolute numbers and rates of incidence of Alzheimer's disease and other dementiase in different age groups in High-income Asia Pacific in 2021

Age	Male (Numbers, 95% UI)	Female (Numbers, 95% UI)	Male (Rates, 95% UI)	Female Rates, 95% UI)
40-44 years	621 (264,1075)	624 (236,1103)	9.28(3.94,16.07)	9.78(3.7,17.28)
45-49 years	2016 (1225,2985)	2331 (1453,3492)	27.12(16.49,40.16)	32.5(20.26,48.68)
50-54 years	3296 (2076,4744)	4152 (2689,5913)	45.7(28.78,65.77)	58.6(37.96,83.46)
55-59 years	5301 (3477,7561)	6792 (4474,9624)	84.67(55.53,120.78)	109.23(71.95,154.77)
60-64 years	9041 (6061,12628)	12004 (8198,16591)	153.76(103.08,214.78)	199.84(136.48,276.21)
65-69 years	15641 (10478,21686)	20687 (13992,29066)	287.12(192.35,398.1)	359.69(243.27,505.37)
70-74 years	31967 (22208,43419)	46813 (32785,63492)	559.38(388.6,759.76)	735.58(515.16,997.65)
75-79 years	40956 (27872,55734)	71005 (49342,95552)	1085(738.39,1476.51)	1518(1054.89,2042.79)
80-84 years	50326 (34425,67362)	101792 (69319,133639)	1803.62(1233.77,2414.2)	2558.04(1742,3358.38)
85-89 years	40219 (28003,54927)	101260 (71189,135169)	2503.88(1743.38,3419.53)	3498.91(2459.83,4670.58)
90-94 years	21268 (14722,29295)	67852 (48614,93019)	3293.87(2280,4536.97)	4376.8(3135.87,6000.22)
95+ years	9862 (6141,14245)	35935 (22472,52038)	4140.96(2578.46,5981.23)	5104.02(3191.76,7391.18)

Table S10.The absolute numbers and rates of YLDs of Alzheimer's disease and other dementiase in different age groups in Non-High-income East Asia in 2021				
**Age**	**Male (Numbers, 95% UI)**	**Female (Numbers, 95% UI)**	**Male (Rates, 95% UI)**	**Female Rates, 95% UI)**
40-44 years	1506 (555,2929)	1873 (706,3461)	3.08(1.13,5.99)	4.03(1.52,7.44)
45-49 years	11981 (6166,21493)	14585 (7494,24885)	20.68(10.64,37.1)	25.98(13.35,44.34)
50-54 years	35059 (20827,55449)	43257 (24669,66973)	55.57(33.01,87.9)	70.09(39.97,108.52)
55-59 years	68331 (40347,101986)	87569 (55010,127956)	120.79(71.32,180.28)	154.04(96.77,225.08)
60-64 years	90771 (57691,135113)	116667 (71404,170517)	238.53(151.61,355.06)	307.35(188.11,449.21)
65-69 years	160413 (100088,229091)	232159 (146663,341621)	412.98(257.68,589.79)	576.57(364.24,848.42)
70-74 years	186272 (115526,276674)	317285 (202869,458467)	700.59(434.51,1040.61)	1117.7(714.65,1615.05)
75-79 years	206605 (135873,293285)	391462 (261668,554203)	1288.07(847.09,1828.47)	2147.23(1435.29,3039.89)
80-84 years	231466 (149698,328496)	479524 (312641,678308)	2581.13(1669.31,3663.14)	4118.95(2685.48,5826.44)
85-89 years	155948 (106929,218502)	405251 (268903,563196)	4319.43(2961.69,6052.02)	6417.39(4258.24,8918.56)
90-94 years	51609 (34904,71916)	190795 (126982,262096)	5932.35(4012.11,8266.7)	8571.12(5704.44,11774.16)
95+ years	10203 (6765,14498)	57982 (39913,79988)	6840.25(4535.14,9719.61)	10471.08(7208.04,14445.1)

Table S11.The absolute numbers and rates of YLDs of Alzheimer's disease and other dementiase in different age groups in Non-High-income Southeast Asia in 2021				
**Age**	**Male (Numbers, 95% UI)**	**Female (Numbers, 95% UI)**	**Male (Rates, 95% UI)**	**Female Rates, 95% UI)**
40-44 years	777 (284,1480)	978 (366,1757)	3.15(1.15,6)	4(1.5,7.19)
45-49 years	4512 (2311,7852)	5633 (2959,9621)	20.4(10.45,35.5)	25.27(13.28,43.16)
50-54 years	10472 (6297,16769)	13579 (7874,21048)	52.74(31.71,84.45)	66.45(38.53,102.99)
55-59 years	18031 (10763,26604)	24989 (16044,36257)	106.54(63.6,157.19)	139.46(89.54,202.35)
60-64 years	26486 (16645,38722)	38833 (23316,56177)	194(121.92,283.63)	260.61(156.47,377)
65-69 years	32093 (19768,46325)	50753 (33289,73283)	326.59(201.16,471.41)	450.99(295.8,651.18)
70-74 years	34240 (21610,50840)	59958 (37929,87852)	556.38(351.15,826.12)	807.41(510.76,1183.04)
75-79 years	33871 (22644,47772)	68113 (46065,96224)	960.53(642.16,1354.75)	1437.65(972.29,2030.98)
80-84 years	34332 (22155,48894)	77337 (50443,108017)	1802.39(1163.1,2566.84)	2646.95(1726.47,3697.03)
85-89 years	25284 (17186,35466)	61766 (40509,85845)	2952.83(2007.02,4141.93)	4190.49(2748.32,5824.11)
90-94 years	12474 (8391,17476)	31290 (20585,43645)	4172.61(2806.84,5845.72)	5804.36(3818.52,8096.24)
95+ years	4712 (3103,6661)	10911 (7559,15007)	5290.88(3483.72,7479.2)	7283.18(5045.86,10017.79)

Table S12.The absolute numbers and rates of YLDs of Alzheimer's disease and other dementiase in different age groups in High-income Asia Pacific in 2021

Age	Male (Numbers, 95% UI)	Female (Numbers, 95% UI)	Male (Rates, 95% UI)	Female Rates, 95% UI)
40-44 years	184 (67,368)	207 (70,413)	2.75(1,5.5)	3.25(1.1,6.47)
45-49 years	1402 (668,2500)	1734 (833,2962)	18.86(8.98,33.64)	24.18(11.61,41.3)
50-54 years	3352 (1911,5266)	4550 (2600,7163)	46.48(26.49,73.01)	64.22(36.7,101.1)
55-59 years	5778 (3462,8583)	8118 (4907,12241)	92.3(55.29,137.1)	130.54(78.91,196.86)
60-64 years	10303 (6599,14959)	15064 (9072,22069)	175.23(112.23,254.42)	250.78(151.03,367.41)
65-69 years	17445 (11131,25187)	26199 (17064,37676)	320.25(204.33,462.36)	455.52(296.69,655.07)
70-74 years	34470 (22469,49859)	54644 (35091,79125)	603.17(393.17,872.46)	858.63(551.38,1243.31)
75-79 years	41283 (27431,59075)	75935 (50655,106729)	1093.68(726.69,1565.02)	1623.4(1082.95,2281.75)
80-84 years	56645 (36744,80277)	123571 (78241,175222)	2030.1(1316.87,2877.05)	3105.38(1966.22,4403.37)
85-89 years	50448 (34172,70507)	142587 (93495,199444)	3140.69(2127.4,4389.45)	4926.89(3230.61,6891.52)
90-94 years	27252 (18174,37938)	103867 (68036,143854)	4220.58(2814.6,5875.53)	6699.97(4388.66,9279.37)
95+ years	12746 (8456,18209)	58601 (39840,83066)	5352.13(3550.47,7645.73)	8323.34(5658.61,11798.29)

Table S13.The ratio of male to female prevalence, incidence, and YLDs rates of Alzheimer's disease and other dementiase according to different age groups in Non-High-income East Asia						
**Age**	**Prevalence**		**Incidence**		**YLDs**	
	**1990**	**2021**	**1990**	**2021**	**1990**	**2021**
40-44 years	0.894468	0.863486	0.893659	0.862744	0.793316	0.764516
45-49 years	0.890626	0.865100	0.881144	0.859703	0.820465	0.795933
50-54 years	0.877511	0.860354	0.862092	0.859675	0.814274	0.792889
55-59 years	0.852279	0.862015	0.815006	0.874159	0.777509	0.784138
60-64 years	0.807246	0.851626	0.747612	0.815726	0.736413	0.776103
65-69 years	0.759659	0.778829	0.706255	0.656580	0.701280	0.716275
70-74 years	0.725689	0.681452	0.694492	0.608004	0.669440	0.626815
75-79 years	0.707448	0.655134	0.692249	0.651776	0.649533	0.599873
80-84 years	0.700493	0.671517	0.724264	0.748738	0.654140	0.626649
85-89 years	0.709605	0.714609	0.765038	0.833922	0.665711	0.673081
90-94 years	0.709017	0.722647	0.782629	0.826025	0.670802	0.692133
95+ years	0.696109	0.666275	0.797824	0.791158	0.669938	0.653251

Table S14.The ratio of male to female prevalence, incidence, and YLDs rates of Alzheimer's disease and other dementiase according to different age groups in Non-High-income Southeast Asia

Age	Prevalence		Incidence		YLDs	
	1990	2021	1990	2021	1990	2021
40-44 years	0.904744	0.887714	0.904281	0.887370	0.801391	0.788038
45-49 years	0.885843	0.874058	0.875243	0.864984	0.816210	0.807162
50-54 years	0.863514	0.855164	0.852021	0.844598	0.797340	0.793701
55-59 years	0.847015	0.837625	0.839259	0.830099	0.765771	0.763912
60-64 years	0.832983	0.819562	0.815356	0.799120	0.752006	0.744408
65-69 years	0.808974	0.790324	0.764355	0.746542	0.739248	0.724161
70-74 years	0.771208	0.752853	0.722076	0.705811	0.703649	0.689090
75-79 years	0.745964	0.732559	0.732774	0.714808	0.680143	0.668125
80-84 years	0.745194	0.732996	0.776687	0.762421	0.692633	0.680934
85-89 years	0.766508	0.751985	0.818916	0.807146	0.717788	0.704649
90-94 years	0.777509	0.762957	0.830979	0.817827	0.735935	0.718875
95+ years	0.780443	0.761883	0.837895	0.828225	0.748168	0.726452

Table S15.The ratio of male to female prevalence, incidence, and YLDs rates of Alzheimer's disease and other dementiase according to different age groups in High-income Asia Pacific						
**Age**	**Prevalence**		**Incidence**		**YLDs**	
	**1990**	**2021**	**1990**	**2021**	**1990**	**2021**
40-44 years	0.780385	0.948917	0.780142	0.948759	0.692136	0.845479
45-49 years	0.791283	0.842826	0.777266	0.834600	0.724719	0.780198
50-54 years	0.771200	0.785585	0.753124	0.779900	0.711440	0.723714
55-59 years	0.752096	0.779003	0.730180	0.775194	0.675010	0.707010
60-64 years	0.730856	0.773490	0.712003	0.769417	0.659049	0.698747
65-69 years	0.728283	0.775426	0.748761	0.798249	0.658597	0.703050
70-74 years	0.748032	0.775298	0.766866	0.760456	0.673454	0.702474
75-79 years	0.757501	0.749291	0.752657	0.714754	0.678380	0.673694
80-84 years	0.747811	0.716651	0.748291	0.705076	0.681878	0.653737
85-89 years	0.734863	0.695971	0.754425	0.715616	0.673369	0.637458
90-94 years	0.723986	0.682644	0.791599	0.752574	0.668742	0.629941
95+ years	0.732669	0.687095	0.846754	0.811313	0.688050	0.643027

Table S16.The SDI and age-standardized YLD rates for Both with Alzheimer's disease and other dementias from 1990 to 2019.												
Year	Global		Non-High-income East Asia		Democratic People's Republic of Korea		China		Taiwan (Province of China)		Non-High-income Southeast Asia	
	SDI	Rate	SDI	Rate	SDI	Rate	SDI	Rate	SDI	Rate	SDI	Rate
1990	0.526	138.3100	0.471	144.6010	0.498	133.4795	0.459	145.7776	0.668	105.4876	0.464	137.2698
1991	0.530	138.8258	0.480	148.7701	0.499	133.5451	0.467	150.1604	0.677	105.1633	0.472	137.1800
1992	0.535	139.2880	0.488	152.5251	0.500	133.5492	0.476	154.1118	0.685	104.8727	0.479	136.9960
1993	0.539	139.6914	0.497	155.7585	0.500	133.6356	0.484	157.5161	0.693	104.4853	0.487	136.8850
1994	0.542	139.9710	0.505	158.1858	0.500	133.6474	0.493	160.0827	0.701	104.3861	0.495	136.7743
1995	0.546	140.1211	0.514	159.5936	0.499	133.8556	0.502	161.5580	0.710	104.0654	0.503	136.6563
1996	0.551	140.1765	0.524	160.3475	0.498	134.0555	0.512	162.3232	0.719	104.1472	0.511	136.5337
1997	0.555	140.2023	0.534	160.9160	0.497	134.2353	0.522	162.9153	0.729	103.9019	0.519	136.3582
1998	0.559	140.1680	0.543	161.3538	0.496	134.5829	0.531	163.3607	0.738	103.8819	0.524	136.1619
1999	0.564	140.1205	0.551	161.6877	0.496	134.8174	0.540	163.7123	0.745	103.8918	0.530	135.9884
2000	0.568	140.0150	0.559	161.8610	0.497	134.9952	0.548	163.9052	0.753	103.8941	0.535	135.8809
2001	0.572	139.9327	0.564	162.1266	0.500	134.9625	0.554	164.2108	0.761	103.7461	0.539	135.8666
2002	0.576	139.8800	0.571	162.4895	0.503	134.7867	0.561	164.6285	0.769	103.6794	0.543	136.0147
2003	0.580	139.8449	0.579	162.8430	0.506	134.5934	0.569	165.0432	0.777	103.6000	0.548	136.1781
2004	0.584	139.8090	0.588	163.1019	0.510	134.2565	0.578	165.3798	0.785	103.7149	0.553	136.3128
2005	0.589	139.7129	0.597	163.1115	0.515	134.2133	0.588	165.4371	0.792	103.8256	0.557	136.3071
2006	0.594	139.4912	0.607	162.8724	0.520	134.0734	0.599	165.1628	0.800	105.5010	0.562	136.1623
2007	0.599	139.1983	0.618	162.4240	0.524	133.8621	0.610	164.5936	0.808	109.0871	0.568	135.9595
2008	0.603	138.8541	0.627	161.8656	0.529	133.7818	0.619	163.8830	0.815	113.2209	0.573	135.7248
2009	0.608	138.5453	0.636	161.3983	0.533	133.6099	0.629	163.2996	0.821	116.5859	0.578	135.4833
2010	0.613	138.3172	0.648	161.1976	0.537	133.3810	0.642	163.0412	0.827	118.2666	0.584	135.2511
2011	0.618	138.1973	0.658	162.0241	0.541	133.0506	0.651	163.9104	0.832	117.9187	0.590	134.9372
2012	0.622	138.2650	0.663	164.2121	0.545	132.7598	0.657	166.2134	0.837	116.8545	0.597	134.5485
2013	0.626	138.4474	0.669	166.8702	0.549	132.4348	0.663	169.0060	0.841	115.6019	0.603	134.1165
2014	0.631	138.7184	0.674	169.2455	0.554	132.1075	0.669	171.5071	0.846	114.3937	0.609	133.7050
2015	0.635	138.8796	0.677	170.5416	0.557	131.8482	0.672	172.8470	0.850	114.0511	0.616	133.3680
2016	0.640	138.9419	0.682	170.7911	0.561	131.7039	0.676	173.0671	0.854	114.2534	0.622	133.0657
2017	0.646	139.0422	0.690	170.7984	0.563	131.5778	0.685	173.0262	0.859	114.6875	0.628	132.7688
2018	0.651	139.1015	0.699	170.7054	0.565	131.5303	0.694	172.8874	0.863	115.0309	0.634	132.4416
2019	0.657	139.0691	0.708	170.6206	0.567	131.5060	0.704	172.7729	0.867	115.1820	0.640	132.1872
2020	0.661	139.1813	0.718	170.3208	0.568	131.0141	0.713	172.4466	0.871	115.2610	0.645	131.9071
2021	0.666	141.9527	0.726	183.0131	0.570	130.8328	0.722	185.6336	0.875	115.3179	0.650	131.3468

Table S16 (continued).												
Year	Thailand		Philippines		Sri Lanka		Timor-Leste		Viet Nam		Mauritius	
	SDI	Rate	SDI	Rate	SDI	Rate	SDI	Rate	SDI	Rate	SDI	Rate
1990	0.507	122.8387	0.510	138.6301	0.523	132.8794	0.262	143.0395	0.408	142.1650	0.545	135.9607
1991	0.516	122.6366	0.514	138.8098	0.528	132.7190	0.271	142.3456	0.413	142.2786	0.548	135.5834
1992	0.525	122.2192	0.517	138.9311	0.533	132.5078	0.280	141.7207	0.420	142.0994	0.553	135.3836
1993	0.534	121.9128	0.521	139.0937	0.539	132.0678	0.289	140.9185	0.427	142.0805	0.561	135.2379
1994	0.543	121.6289	0.525	139.2654	0.545	131.7967	0.299	140.2135	0.435	142.0726	0.569	134.7732
1995	0.552	121.4671	0.529	139.4621	0.552	131.6342	0.308	139.6187	0.444	142.0061	0.577	134.5568
1996	0.561	121.1107	0.533	139.6115	0.559	131.3804	0.318	138.9317	0.453	141.9160	0.585	134.1712
1997	0.568	120.6577	0.537	139.7730	0.566	130.9932	0.327	138.0550	0.463	141.7538	0.591	133.7332
1998	0.574	120.3862	0.539	139.9180	0.572	130.4329	0.335	137.2194	0.472	141.4939	0.597	133.1422
1999	0.579	120.1331	0.542	140.0448	0.578	130.0810	0.339	136.5253	0.481	141.2491	0.602	132.5962
2000	0.584	120.1385	0.545	140.1492	0.583	129.7587	0.344	135.9405	0.490	141.0694	0.607	132.2412
2001	0.588	120.8435	0.547	140.1763	0.588	129.6035	0.349	135.5923	0.498	140.7478	0.612	132.0977
2002	0.592	122.6537	0.550	140.0876	0.592	129.3998	0.354	135.6114	0.505	140.2982	0.617	131.9534
2003	0.596	124.8879	0.552	139.9586	0.597	129.2755	0.358	135.6403	0.513	139.7488	0.623	131.8250
2004	0.601	126.7370	0.554	139.7864	0.602	129.0478	0.363	135.7725	0.521	139.2808	0.629	131.7898
2005	0.605	127.6666	0.556	139.5532	0.607	128.6658	0.367	135.7670	0.529	138.8821	0.635	131.8509
2006	0.610	128.0639	0.558	139.2689	0.612	128.4510	0.371	135.6320	0.536	138.5115	0.640	132.0837
2007	0.616	128.2502	0.561	138.8352	0.617	128.2613	0.375	135.3687	0.543	138.1172	0.644	132.4217
2008	0.621	128.2832	0.564	138.3551	0.622	128.1509	0.381	135.3916	0.550	137.6679	0.650	132.8423
2009	0.624	128.5911	0.567	137.9052	0.628	127.9554	0.388	135.2386	0.556	137.0812	0.655	133.3093
2010	0.629	128.4624	0.572	137.5727	0.634	127.8560	0.395	135.1347	0.563	136.7423	0.660	133.3719
2011	0.633	128.1918	0.577	137.2833	0.641	127.7089	0.402	135.0649	0.569	136.4414	0.666	133.4116
2012	0.638	127.6547	0.584	136.8987	0.648	127.5684	0.409	134.9793	0.575	136.1201	0.673	133.3218
2013	0.644	127.0294	0.591	136.4699	0.655	127.4131	0.415	134.9826	0.581	135.9290	0.680	133.2624
2014	0.649	126.2968	0.598	136.1138	0.663	127.3850	0.421	134.7667	0.587	135.6618	0.687	133.1881
2015	0.654	125.9175	0.606	135.8724	0.670	127.3795	0.426	134.8556	0.593	135.4394	0.692	133.1126
2016	0.659	125.7171	0.614	135.6698	0.676	127.3727	0.431	134.5548	0.599	135.2584	0.697	133.0167
2017	0.665	125.7074	0.622	135.4054	0.682	127.5064	0.435	134.2625	0.605	135.0309	0.702	132.9573
2018	0.670	125.5873	0.630	135.1608	0.688	127.3876	0.437	133.8719	0.611	134.9101	0.706	132.8345
2019	0.675	125.6196	0.639	134.9830	0.694	127.4875	0.440	133.6767	0.618	134.5827	0.711	132.9350
2020	0.679	125.2313	0.645	134.9048	0.698	127.0821	0.442	133.7131	0.623	134.1928	0.715	132.7393
2021	0.683	123.5013	0.651	134.7103	0.702	127.2848	0.445	133.4991	0.628	134.0844	0.718	132.5815

Table S16 (continued).												
Year	Seychelles		High-income Asia Pacific		Singapore		Japan		Republic of Korea		Brunei Darussalam	
	SDI	Rate	SDI	Rate	SDI	Rate	SDI	Rate	SDI	Rate	SDI	Rate
1990	0.576	138.3342	0.768	137.3938	0.686	108.2723	0.790	135.7699	0.692	156.0084	0.666	119.2605
1991	0.583	138.4667	0.773	137.5670	0.695	108.1092	0.795	135.8929	0.703	155.9273	0.671	119.2738
1992	0.590	138.2724	0.779	137.8226	0.703	107.9921	0.799	136.0946	0.712	156.1069	0.677	119.2856
1993	0.598	138.2784	0.784	138.0868	0.712	107.8204	0.803	136.3309	0.722	156.1244	0.682	119.2326
1994	0.606	138.2723	0.789	138.4514	0.721	107.6287	0.807	136.6439	0.732	156.4968	0.687	119.1881
1995	0.614	138.0575	0.794	138.7487	0.730	107.6407	0.811	136.9164	0.742	156.7102	0.692	118.9438
1996	0.622	137.9514	0.799	139.2705	0.739	107.3442	0.814	137.4010	0.752	157.2007	0.698	118.5539
1997	0.631	137.5895	0.803	140.0021	0.748	107.2439	0.817	138.1148	0.761	157.8241	0.703	118.0524
1998	0.638	137.1615	0.807	140.9167	0.755	106.8869	0.819	139.0260	0.768	158.6028	0.709	117.4583
1999	0.645	136.8736	0.810	141.8444	0.761	106.7456	0.821	139.9835	0.777	159.1223	0.715	116.8704
2000	0.652	136.5764	0.814	142.7064	0.768	106.6924	0.823	140.8934	0.785	159.4639	0.722	116.5366
2001	0.656	136.1276	0.818	143.7293	0.774	107.0726	0.825	142.0029	0.793	159.4109	0.729	116.4244
2002	0.661	135.7319	0.821	144.9559	0.780	107.6176	0.827	143.3163	0.801	159.4520	0.735	116.2896
2003	0.664	135.3740	0.825	146.2226	0.784	108.5016	0.829	144.6742	0.808	159.5105	0.742	116.3130
2004	0.666	134.9375	0.828	147.1342	0.790	108.9983	0.832	145.6549	0.814	159.4528	0.747	116.6343
2005	0.669	134.7356	0.831	147.5599	0.796	109.4063	0.834	146.0767	0.819	159.5718	0.752	116.9709
2006	0.672	134.5048	0.834	147.3593	0.803	109.4678	0.836	145.8201	0.824	159.6139	0.757	117.0929
2007	0.675	134.3018	0.837	146.8102	0.811	109.4450	0.838	145.1389	0.829	159.8973	0.762	117.1272
2008	0.677	134.0332	0.839	146.1813	0.818	109.3247	0.840	144.3827	0.834	160.2578	0.766	117.1009
2009	0.678	133.8332	0.842	145.6712	0.824	109.1856	0.842	143.7805	0.838	160.3922	0.770	116.9849
2010	0.680	133.5617	0.845	145.5568	0.830	109.1861	0.844	143.5798	0.843	160.6388	0.773	117.0476
2011	0.684	133.2813	0.848	146.1013	0.834	109.1084	0.847	144.2224	0.847	160.4687	0.777	117.3059
2012	0.688	132.8645	0.851	147.2680	0.837	108.9771	0.849	145.6112	0.851	160.3435	0.780	117.6150
2013	0.693	132.4569	0.854	148.5618	0.840	108.7840	0.851	147.1734	0.855	160.1076	0.784	118.1762
2014	0.697	132.1822	0.857	149.5961	0.843	108.5647	0.854	148.4493	0.860	159.6935	0.788	118.6995
2015	0.702	131.7688	0.860	150.0141	0.845	108.5603	0.856	148.9658	0.864	159.3922	0.791	119.2830
2016	0.706	131.5194	0.863	149.9035	0.848	109.3069	0.859	148.8938	0.868	158.5130	0.795	119.6357
2017	0.711	131.2084	0.866	149.6760	0.849	110.8363	0.861	148.8119	0.873	156.5518	0.798	119.6843
2018	0.716	130.8696	0.869	149.3589	0.851	112.4203	0.864	148.6540	0.877	154.4827	0.801	119.6475
2019	0.722	130.6422	0.872	149.1380	0.852	113.1011	0.867	148.4663	0.881	153.4836	0.804	119.2370
2020	0.727	130.5320	0.874	148.7661	0.854	112.9342	0.869	147.9874	0.884	153.4877	0.807	119.1936
2021	0.730	130.1623	0.877	143.0968	0.856	112.6442	0.871	141.2451	0.887	152.7632	0.810	118.7472

Table S17.The SDI and age-standardized YLD rates for Male with Alzheimer's disease and other dementias from 1990 to 2019.												
Year	Global		East Asia		Democratic People's Republic of Korea		China		Taiwan (Province of China)		Southeast Asia	
	SDI	Rate	SDI	Rate	SDI	Rate	SDI	Rate	SDI	Rate	SDI	Rate
1990	0.526	110.9669	0.471	112.0700	0.498	97.4374	0.459	113.1805	0.668	81.2969	0.464	111.8629
1991	0.530	111.2754	0.480	114.8416	0.499	97.3820	0.467	116.1015	0.677	81.2313	0.472	111.7332
1992	0.535	111.5107	0.488	117.3404	0.500	97.2404	0.476	118.7443	0.685	81.2487	0.479	111.5043
1993	0.539	111.7053	0.497	119.5404	0.500	97.2906	0.484	121.0721	0.693	81.0919	0.487	111.3953
1994	0.542	111.8209	0.505	121.2447	0.500	97.2432	0.493	122.8883	0.701	81.2410	0.495	111.2921
1995	0.546	111.8571	0.514	122.3493	0.499	97.3238	0.502	124.0590	0.710	81.0821	0.503	111.1866
1996	0.551	111.7998	0.524	123.0843	0.498	97.3800	0.512	124.8191	0.719	81.1615	0.511	111.1372
1997	0.555	111.6581	0.534	123.6389	0.497	97.4385	0.522	125.4152	0.729	80.9764	0.519	111.0847
1998	0.559	111.4663	0.543	124.0797	0.496	97.6509	0.531	125.8829	0.738	80.9132	0.524	110.9902
1999	0.564	111.3047	0.551	124.4564	0.496	97.6609	0.540	126.2988	0.745	80.9195	0.530	110.8918
2000	0.568	111.1769	0.559	124.6157	0.497	97.6606	0.548	126.4929	0.753	80.9304	0.535	110.8196
2001	0.572	111.1466	0.564	124.8828	0.500	97.5714	0.554	126.8189	0.761	80.7314	0.539	110.7978
2002	0.576	111.1766	0.571	125.3310	0.503	97.5038	0.561	127.3456	0.769	80.7060	0.543	110.8777
2003	0.580	111.2477	0.579	125.7539	0.506	97.4176	0.569	127.8619	0.777	80.5212	0.548	110.9849
2004	0.584	111.3026	0.588	126.0866	0.510	97.5106	0.578	128.2988	0.785	80.6147	0.553	110.9855
2005	0.589	111.3305	0.597	126.1942	0.515	97.4902	0.588	128.4781	0.792	80.6469	0.557	110.8965
2006	0.594	111.3061	0.607	126.1653	0.520	97.3215	0.599	128.4436	0.800	81.5891	0.562	110.7092
2007	0.599	111.2846	0.618	126.1103	0.524	97.1286	0.610	128.3168	0.808	83.4938	0.568	110.4045
2008	0.603	111.2271	0.627	125.9771	0.529	96.9832	0.619	128.0794	0.815	85.6588	0.573	110.0445
2009	0.608	111.1782	0.636	125.8788	0.533	96.7864	0.629	127.9014	0.821	87.5114	0.578	109.6979
2010	0.613	111.1630	0.648	125.9576	0.537	96.6305	0.642	127.9314	0.827	88.3307	0.584	109.3983
2011	0.618	111.1923	0.658	126.8096	0.541	96.4897	0.651	128.8141	0.832	87.8669	0.590	109.0583
2012	0.622	111.3656	0.663	128.7169	0.545	96.3076	0.657	130.8172	0.837	86.9218	0.597	108.6641
2013	0.626	111.6156	0.669	130.9324	0.549	96.1007	0.663	133.1450	0.841	85.7659	0.603	108.1973
2014	0.631	111.9451	0.674	132.8329	0.554	96.0028	0.669	135.1487	0.846	84.7863	0.609	107.8324
2015	0.635	112.1481	0.677	133.7742	0.557	95.8970	0.672	136.1072	0.850	84.2648	0.616	107.4292
2016	0.640	112.2014	0.682	133.8336	0.561	95.9052	0.676	136.1086	0.854	84.2731	0.622	107.0578
2017	0.646	112.2120	0.690	133.6853	0.563	95.7985	0.685	135.8900	0.859	84.3532	0.628	106.6150
2018	0.651	112.1892	0.699	133.4701	0.565	95.6927	0.694	135.6171	0.863	84.3101	0.634	106.1595
2019	0.657	112.1495	0.708	133.3248	0.567	95.6941	0.704	135.4367	0.867	84.3377	0.640	105.8534
2020	0.661	112.3830	0.718	133.1835	0.568	96.1024	0.713	135.2559	0.871	84.4109	0.645	105.6675
2021	0.666	114.3844	0.726	141.3056	0.570	95.6657	0.722	143.6956	0.875	84.1201	0.650	105.4543

Table S17 (continued).												
Year	Indonesia		Cambodia		Lao People's Democratic Republic		Maldives		Malaysia		Myanmar	
	SDI	Rate	SDI	Rate	SDI	Rate	SDI	Rate	SDI	Rate	SDI	Rate
1990	0.457	114.4831	0.289	112.4377	0.264	113.2605	0.332	115.6240	0.546	109.9127	0.319	116.5143
1991	0.467	114.4249	0.292	112.6715	0.269	112.8744	0.344	115.2933	0.552	109.8282	0.322	116.0189
1992	0.476	114.3579	0.297	112.6493	0.273	112.4537	0.358	115.0961	0.558	109.7567	0.326	115.1935
1993	0.485	114.3296	0.302	112.7686	0.278	112.0284	0.373	114.8730	0.566	109.7351	0.330	114.7915
1994	0.494	114.3669	0.306	112.6969	0.283	111.8646	0.388	114.5280	0.574	109.4765	0.334	114.3233
1995	0.502	114.3846	0.311	112.8376	0.289	111.7148	0.404	114.4454	0.583	109.4478	0.340	113.8802
1996	0.510	114.4558	0.316	112.8467	0.295	111.7504	0.421	114.2883	0.593	109.4471	0.345	113.6773
1997	0.518	114.5420	0.321	112.9350	0.301	111.7886	0.437	114.0572	0.603	109.3255	0.351	113.5009
1998	0.524	114.5875	0.325	113.2049	0.308	111.7123	0.454	113.7954	0.613	109.3332	0.357	113.3229
1999	0.529	114.6567	0.330	113.3499	0.316	111.7742	0.469	113.4563	0.622	109.1140	0.363	112.9134
2000	0.534	114.6783	0.336	113.2542	0.324	111.7661	0.483	113.3573	0.631	109.0828	0.370	112.8893
2001	0.538	114.7753	0.343	112.9279	0.333	111.8258	0.496	113.1560	0.638	108.9737	0.378	112.4038
2002	0.542	114.8558	0.349	112.8959	0.342	111.6785	0.507	112.9998	0.645	108.9568	0.386	112.0599
2003	0.547	114.9646	0.357	112.4146	0.351	111.6308	0.519	112.8207	0.651	108.8290	0.395	111.6568
2004	0.551	114.9615	0.365	112.0628	0.360	111.4176	0.530	112.7475	0.656	108.7550	0.405	111.1228
2005	0.555	114.9806	0.373	111.5291	0.369	111.2300	0.538	112.4740	0.661	108.5165	0.415	110.7529
2006	0.560	114.8819	0.382	111.0813	0.378	111.3587	0.548	112.3623	0.666	108.5807	0.425	110.4634
2007	0.566	114.6586	0.390	110.6803	0.388	111.2834	0.558	112.3625	0.671	108.5641	0.435	109.9397
2008	0.572	114.4231	0.398	110.1066	0.397	111.3592	0.567	112.2619	0.676	108.5422	0.444	109.4223
2009	0.579	114.1874	0.404	109.5040	0.406	111.2634	0.574	112.3478	0.682	108.3792	0.452	108.9675
2010	0.586	113.9692	0.410	109.1617	0.415	111.2578	0.582	112.4670	0.688	108.5442	0.460	108.4446
2011	0.594	113.6722	0.416	108.8624	0.423	111.1500	0.590	112.3626	0.694	108.3893	0.468	108.3039
2012	0.601	113.2992	0.422	108.6080	0.431	110.9496	0.597	112.7044	0.700	108.5514	0.475	107.9938
2013	0.609	112.8681	0.428	108.2458	0.439	110.7694	0.604	112.7729	0.705	108.6598	0.483	107.6721
2014	0.615	112.4522	0.434	108.0381	0.446	110.7484	0.612	112.8831	0.710	108.8489	0.490	107.5603
2015	0.622	112.0036	0.439	107.8473	0.453	110.6564	0.618	112.9103	0.715	108.9635	0.497	107.4561
2016	0.629	111.6432	0.445	107.4946	0.460	110.5528	0.625	112.8622	0.719	108.5988	0.504	107.0406
2017	0.635	111.1455	0.451	107.1263	0.466	110.3885	0.631	112.9543	0.724	108.1938	0.511	106.5797
2018	0.641	110.5821	0.457	106.7861	0.473	110.1700	0.637	113.0623	0.729	107.7290	0.518	106.1204
2019	0.647	110.1983	0.464	106.5987	0.479	109.9750	0.643	113.1015	0.735	107.2812	0.524	105.8944
2020	0.652	110.1247	0.469	106.2618	0.484	109.8352	0.648	113.2563	0.739	107.4066	0.529	105.8127
2021	0.657	110.2337	0.474	106.2690	0.489	110.0315	0.651	113.1015	0.743	105.3605	0.534	105.4877

Table S17 (continued).												
Year	Thailand		Philippines		Sri Lanka		Timor-Leste		Viet Nam		Mauritius	
	SDI	Rate	SDI	Rate	SDI	Rate	SDI	Rate	SDI	Rate	SDI	Rate
1990	0.507	97.6158	0.510	116.3764	0.523	116.0674	0.262	119.7290	0.408	111.8960	0.545	108.3509
1991	0.516	97.5787	0.514	116.2803	0.528	115.4785	0.271	119.2353	0.413	112.0325	0.548	107.9855
1992	0.525	97.2770	0.517	116.1978	0.533	114.9881	0.280	118.9254	0.420	111.8312	0.553	107.7017
1993	0.534	97.1485	0.521	116.1448	0.539	114.3365	0.289	118.4144	0.427	111.8418	0.561	107.4970
1994	0.543	97.0100	0.525	116.1252	0.545	113.8226	0.299	117.8679	0.435	111.7817	0.569	107.2766
1995	0.552	96.9519	0.529	116.1076	0.552	113.5089	0.308	117.5607	0.444	111.5070	0.577	106.9779
1996	0.561	96.7108	0.533	116.1039	0.559	113.3107	0.318	117.1989	0.453	111.5434	0.585	106.6884
1997	0.568	96.4884	0.537	116.2063	0.566	112.8863	0.327	116.6386	0.463	111.5373	0.591	106.2849
1998	0.574	96.3027	0.539	116.2704	0.572	112.3973	0.335	116.0888	0.472	111.3412	0.597	105.8153
1999	0.579	96.0909	0.542	116.3541	0.578	112.2310	0.339	115.6044	0.481	111.1018	0.602	105.4892
2000	0.584	96.1062	0.545	116.3759	0.583	111.8209	0.344	115.1656	0.490	110.9140	0.607	105.0873
2001	0.588	96.6780	0.547	116.3459	0.588	111.5110	0.349	115.0110	0.498	110.7107	0.612	105.0239
2002	0.592	98.0601	0.550	116.2242	0.592	111.1679	0.354	115.0735	0.505	110.2480	0.617	105.0402
2003	0.596	99.6433	0.552	116.0696	0.597	110.7625	0.358	115.1328	0.513	109.8268	0.623	104.9553
2004	0.601	100.8899	0.554	115.8920	0.602	110.3085	0.363	115.1279	0.521	109.3560	0.629	104.9458
2005	0.605	101.6170	0.556	115.6419	0.607	109.8292	0.367	115.1220	0.529	108.8993	0.635	105.0831
2006	0.610	101.7616	0.558	115.3231	0.612	109.5418	0.371	114.8442	0.536	108.6339	0.640	105.1434
2007	0.616	101.9060	0.561	114.8507	0.617	109.0101	0.375	114.3872	0.543	108.2100	0.644	105.3698
2008	0.621	102.0069	0.564	114.3121	0.622	108.6302	0.381	114.3012	0.550	107.5097	0.650	105.7392
2009	0.624	102.1155	0.567	113.7944	0.628	108.3819	0.388	113.9036	0.556	106.9287	0.655	105.9654
2010	0.629	102.0737	0.572	113.3195	0.634	108.1668	0.395	113.6382	0.563	106.6737	0.660	106.0831
2011	0.633	101.8911	0.577	112.8772	0.641	107.8825	0.402	113.4105	0.569	106.3982	0.666	106.1391
2012	0.638	101.5927	0.584	112.3259	0.648	107.8112	0.409	113.2661	0.575	106.0618	0.673	106.0400
2013	0.644	101.0072	0.591	111.7548	0.655	107.6565	0.415	113.2271	0.581	105.7916	0.680	105.9532
2014	0.649	100.5339	0.598	111.2485	0.663	107.5145	0.421	112.9508	0.587	105.8024	0.687	106.1105
2015	0.654	100.1877	0.606	110.8625	0.670	107.3584	0.426	112.7865	0.593	105.4260	0.692	106.0004
2016	0.659	99.8850	0.614	110.5137	0.676	107.2820	0.431	112.3889	0.599	105.2193	0.697	105.8402
2017	0.665	99.6146	0.622	110.1455	0.682	107.2140	0.435	111.7071	0.605	104.8540	0.702	105.7384
2018	0.670	99.4936	0.630	109.8024	0.688	106.9306	0.437	111.0479	0.611	104.4314	0.706	105.6013
2019	0.675	99.4464	0.639	109.5151	0.694	106.9434	0.440	110.6650	0.618	104.1685	0.711	105.5869
2020	0.679	99.3792	0.645	109.2921	0.698	106.1774	0.442	110.4769	0.623	103.9344	0.715	105.4891
2021	0.683	98.4050	0.651	109.0051	0.702	106.6723	0.445	110.3243	0.628	104.1094	0.718	105.5322

Table S17 (continued).												
Year	Seychelles		High-income Asia Pacific		Singapore		Japan		Republic of Korea		Brunei Darussalam	
	SDI	Rate	SDI	Rate	SDI	Rate	SDI	Rate	SDI	Rate	SDI	Rate
1990	0.576	107.5331	0.768	105.4759	0.686	89.1067	0.790	104.8667	0.692	113.7190	0.666	99.1448
1991	0.583	107.4996	0.773	105.6114	0.695	88.9442	0.795	105.0014	0.703	113.2672	0.671	99.0899
1992	0.590	107.2704	0.779	105.7632	0.703	88.8443	0.799	105.1423	0.712	113.1438	0.677	98.9775
1993	0.598	106.9060	0.784	105.9137	0.712	88.9151	0.803	105.2737	0.722	113.1170	0.682	98.6299
1994	0.606	106.9296	0.789	106.1331	0.721	88.7001	0.807	105.4491	0.732	113.4871	0.687	98.4963
1995	0.614	106.7643	0.794	106.3153	0.730	88.6982	0.811	105.5938	0.742	113.8822	0.692	98.2280
1996	0.622	106.5388	0.799	106.5829	0.739	88.4083	0.814	105.7463	0.752	115.1260	0.698	97.9969
1997	0.631	106.2404	0.803	106.8932	0.748	88.4491	0.817	105.9098	0.761	116.9605	0.703	97.6967
1998	0.638	106.1140	0.807	107.2389	0.755	88.0778	0.819	106.0454	0.768	119.3337	0.709	97.3810
1999	0.645	106.0728	0.810	107.5237	0.761	87.9078	0.821	106.1688	0.777	121.1307	0.715	97.0395
2000	0.652	105.9551	0.814	107.8179	0.768	87.8684	0.823	106.3848	0.785	122.0953	0.722	97.0006
2001	0.656	105.9459	0.818	108.1131	0.774	88.2769	0.825	106.6620	0.793	122.1990	0.729	97.1451
2002	0.661	105.7380	0.821	108.4458	0.780	88.5506	0.827	106.9905	0.801	122.1693	0.735	97.2220
2003	0.664	105.6633	0.825	108.8156	0.784	89.3473	0.829	107.3644	0.808	122.0821	0.742	97.4992
2004	0.666	105.6805	0.828	109.1675	0.790	89.8180	0.832	107.7054	0.814	122.1290	0.747	97.8442
2005	0.669	105.5423	0.831	109.4354	0.796	90.1598	0.834	107.9920	0.819	122.0404	0.752	98.0062
2006	0.672	105.2580	0.834	109.7959	0.803	90.1949	0.836	108.3870	0.824	122.0824	0.757	98.0639
2007	0.675	105.2710	0.837	110.3779	0.811	90.3641	0.838	108.9864	0.829	122.4112	0.762	97.9110
2008	0.677	104.9146	0.839	111.0453	0.818	90.2690	0.840	109.7146	0.834	122.7460	0.766	97.9603
2009	0.678	104.5788	0.842	111.7014	0.824	90.3204	0.842	110.4892	0.838	122.7420	0.770	97.9789
2010	0.680	104.5138	0.845	112.2467	0.830	90.3392	0.844	111.0759	0.843	122.9576	0.773	97.8776
2011	0.684	104.3528	0.848	112.8562	0.834	90.3364	0.847	111.7916	0.847	122.8644	0.777	98.0006
2012	0.688	104.0762	0.851	113.6691	0.837	90.3296	0.849	112.7295	0.851	122.8176	0.780	98.0098
2013	0.693	103.8946	0.854	114.4377	0.840	90.2945	0.851	113.6431	0.855	122.6369	0.784	98.0065
2014	0.697	103.7150	0.857	115.0213	0.843	90.2484	0.854	114.3336	0.860	122.4071	0.788	98.0138
2015	0.702	103.5366	0.860	115.3168	0.845	90.3088	0.856	114.6330	0.864	122.4337	0.791	98.0099
2016	0.706	103.3452	0.863	115.3619	0.848	90.8653	0.859	114.5741	0.868	122.8173	0.795	97.9266
2017	0.711	103.1052	0.866	115.4100	0.849	92.0757	0.861	114.4400	0.873	123.3597	0.798	97.5324
2018	0.716	102.7864	0.869	115.3765	0.851	93.2195	0.864	114.2407	0.877	123.7889	0.801	97.3226
2019	0.722	102.5681	0.872	115.3548	0.852	93.6731	0.867	114.0782	0.881	124.1225	0.804	97.0872
2020	0.727	102.4655	0.874	115.1837	0.854	93.4093	0.869	113.7632	0.884	124.3983	0.807	97.0344
2021	0.730	102.4314	0.877	110.5256	0.856	93.5775	0.871	108.1824	0.887	124.0526	0.810	96.8998

Table S18.The SDI and age-standardized YLD rates for Female with Alzheimer's disease and other dementias from 1990 to 2019.												
Year	Global		East Asia		Democratic People's Republic of Korea		China		Taiwan (Province of China)		Southeast Asia	
	SDI	Rate	SDI	Rate	SDI	Rate	SDI	Rate	SDI	Rate	SDI	Rate
1990	0.526	155.6007	0.471	165.3507	0.498	148.0767	0.459	166.6109	0.668	126.0843	0.464	155.9839
1991	0.530	156.2931	0.480	170.6016	0.499	148.0714	0.467	172.1139	0.677	125.8843	0.472	155.9631
1992	0.535	156.9604	0.488	175.3625	0.500	148.0184	0.476	177.1001	0.685	125.8045	0.479	155.8519
1993	0.539	157.5540	0.497	179.4134	0.500	147.9737	0.484	181.3461	0.693	125.5643	0.487	155.7672
1994	0.542	157.9953	0.505	182.4399	0.500	147.8810	0.493	184.5243	0.701	125.5858	0.495	155.6695
1995	0.546	158.2853	0.514	184.2204	0.499	147.8689	0.502	186.3741	0.710	125.3922	0.503	155.5507
1996	0.551	158.4827	0.524	185.1529	0.498	147.7455	0.512	187.3111	0.719	125.7226	0.511	155.3756
1997	0.555	158.6949	0.534	185.8841	0.497	147.6993	0.522	188.0544	0.729	125.7073	0.519	155.0949
1998	0.559	158.8413	0.543	186.4227	0.496	147.8623	0.531	188.5887	0.738	125.9162	0.524	154.7948
1999	0.564	158.9390	0.551	186.7905	0.496	148.0569	0.540	188.9554	0.745	126.1426	0.530	154.5220
2000	0.568	158.9291	0.559	187.0460	0.497	148.1562	0.548	189.2180	0.753	126.3122	0.535	154.3402
2001	0.572	158.8887	0.564	187.3311	0.500	148.0317	0.554	189.5277	0.761	126.3268	0.539	154.2831
2002	0.576	158.8685	0.571	187.6602	0.503	147.7174	0.561	189.8953	0.769	126.2968	0.543	154.4227
2003	0.580	158.8392	0.579	187.9193	0.506	147.4407	0.569	190.1961	0.777	126.3452	0.548	154.5633
2004	0.584	158.8123	0.588	188.0546	0.510	146.8872	0.578	190.3899	0.785	126.5794	0.553	154.7266
2005	0.589	158.7201	0.597	188.0279	0.515	146.8786	0.588	190.3968	0.792	126.7358	0.557	154.7053
2006	0.594	158.4680	0.607	187.7586	0.520	146.7619	0.599	190.0825	0.800	129.0145	0.562	154.5359
2007	0.599	158.1094	0.618	187.2073	0.524	146.5678	0.610	189.3907	0.808	133.9997	0.568	154.3415
2008	0.603	157.6867	0.627	186.5471	0.529	146.5362	0.619	188.5583	0.815	139.7428	0.573	154.1168
2009	0.608	157.3123	0.636	186.0256	0.533	146.4548	0.629	187.8985	0.821	144.4357	0.578	153.8721
2010	0.613	157.0434	0.648	185.8364	0.537	146.2340	0.642	187.6523	0.827	146.6115	0.584	153.6115
2011	0.618	156.9285	0.658	186.8825	0.541	145.9268	0.651	188.7686	0.832	145.9245	0.590	153.2900
2012	0.622	157.0629	0.663	189.5299	0.545	145.7240	0.657	191.5773	0.837	144.2165	0.597	152.8682
2013	0.626	157.3321	0.669	192.7224	0.549	145.4909	0.663	194.9540	0.841	142.3303	0.603	152.4442
2014	0.631	157.6719	0.674	195.5971	0.554	145.1626	0.669	197.9923	0.846	140.6519	0.609	151.9926
2015	0.635	157.8981	0.677	197.2138	0.557	145.0156	0.672	199.6870	0.850	139.9397	0.616	151.7032
2016	0.640	158.0481	0.682	197.6080	0.561	144.9922	0.676	200.0812	0.854	139.7299	0.622	151.5010
2017	0.646	158.2793	0.690	197.6915	0.563	145.0927	0.685	200.1403	0.859	139.8787	0.628	151.3306
2018	0.651	158.4654	0.699	197.6380	0.565	145.3674	0.694	200.0570	0.863	140.0115	0.634	151.1217
2019	0.657	158.5206	0.708	197.5840	0.567	145.5309	0.704	199.9773	0.867	140.0665	0.640	150.9209
2020	0.661	158.6016	0.718	197.1550	0.568	144.9006	0.713	199.5398	0.871	139.7115	0.645	150.5667
2021	0.666	161.9217	0.726	213.3223	0.570	145.0966	0.722	216.3775	0.875	139.6201	0.650	149.6926

Table S18 (continued).												
Year	Indonesia		Cambodia		Lao People's Democratic Republic		Maldives		Malaysia		Myanmar	
	SDI	Rate	SDI	Rate	SDI	Rate	SDI	Rate	SDI	Rate	SDI	Rate
1990	0.457	156.4236	0.289	154.3236	0.264	156.3255	0.332	159.8933	0.546	168.0585	0.319	167.4843
1991	0.467	156.6151	0.292	154.5285	0.269	156.1940	0.344	159.9237	0.552	168.4072	0.322	166.7772
1992	0.476	156.7782	0.297	154.8213	0.273	155.9208	0.358	159.7398	0.558	168.5552	0.326	166.0963
1993	0.485	156.9255	0.302	155.1463	0.278	155.8224	0.373	159.5651	0.566	168.7807	0.330	165.4481
1994	0.494	156.9623	0.306	155.3377	0.283	155.7089	0.388	159.4014	0.574	168.9597	0.334	164.9186
1995	0.502	156.9573	0.311	155.2221	0.289	155.4342	0.404	159.2144	0.583	169.0074	0.340	164.1281
1996	0.510	156.9675	0.316	155.2058	0.295	155.3466	0.421	158.9378	0.593	168.9356	0.345	163.7665
1997	0.518	156.9070	0.321	155.1874	0.301	155.1025	0.437	158.6185	0.603	168.8353	0.351	163.1527
1998	0.524	156.8378	0.325	155.0974	0.308	154.7548	0.454	158.2464	0.613	168.4710	0.357	162.4302
1999	0.529	156.7210	0.330	155.1290	0.316	154.4560	0.469	157.6232	0.622	168.1803	0.363	161.9694
2000	0.534	156.6049	0.336	154.9880	0.324	154.3116	0.483	157.4528	0.631	168.2837	0.370	161.5339
2001	0.538	156.6374	0.343	154.5916	0.333	154.2467	0.496	157.1028	0.638	168.0361	0.378	161.2111
2002	0.542	156.7466	0.349	154.5337	0.342	154.0799	0.507	156.9497	0.645	167.6196	0.386	160.6195
2003	0.547	156.6780	0.357	154.0473	0.351	154.0139	0.519	156.6384	0.651	167.1635	0.395	160.0901
2004	0.551	156.7218	0.365	153.8642	0.360	153.9624	0.530	156.4286	0.656	166.9059	0.405	159.6467
2005	0.555	156.7823	0.373	153.4726	0.369	153.9208	0.538	156.2853	0.661	166.5850	0.415	159.2374
2006	0.560	156.6906	0.382	153.2682	0.378	153.9658	0.548	156.3924	0.666	166.3261	0.425	158.7549
2007	0.566	156.7056	0.390	153.0437	0.388	153.9121	0.558	156.7760	0.671	166.0188	0.435	158.0875
2008	0.572	156.7144	0.398	152.6959	0.397	154.2658	0.567	156.8931	0.676	165.7301	0.444	157.5024
2009	0.579	156.6397	0.404	152.4775	0.406	154.3976	0.574	157.1035	0.682	165.6026	0.452	156.7068
2010	0.586	156.5024	0.410	152.1797	0.415	154.4415	0.582	157.6076	0.688	165.4914	0.460	156.5691
2011	0.594	156.2947	0.416	151.9153	0.423	154.5045	0.590	157.6987	0.694	165.4971	0.468	156.1089
2012	0.601	155.9791	0.422	151.2809	0.431	154.3979	0.597	158.2883	0.700	165.6824	0.475	155.7843
2013	0.609	155.5007	0.428	150.9064	0.439	154.4648	0.604	158.8351	0.705	166.1204	0.483	155.4751
2014	0.615	155.1792	0.434	150.4305	0.446	154.3431	0.612	159.3323	0.710	166.3794	0.490	155.2522
2015	0.622	154.8479	0.439	150.2949	0.453	154.3887	0.618	159.6836	0.715	166.4870	0.497	154.9259
2016	0.629	154.6514	0.445	149.9627	0.460	154.2510	0.625	159.9671	0.719	166.2069	0.504	154.8035
2017	0.635	154.3640	0.451	149.8416	0.466	154.2904	0.631	160.3205	0.724	165.7896	0.511	154.5754
2018	0.641	154.0277	0.457	149.8012	0.473	154.3969	0.637	160.6660	0.729	165.4187	0.518	154.3970
2019	0.647	153.7858	0.464	149.6627	0.479	154.4059	0.643	160.8646	0.735	165.0800	0.524	154.1666
2020	0.652	153.7148	0.469	149.3728	0.484	154.1317	0.648	160.8888	0.739	164.6505	0.529	154.0505
2021	0.657	153.5152	0.474	149.1801	0.489	153.9372	0.651	160.7085	0.743	159.4157	0.534	153.0779

Table S18 (continued).												
Year	Thailand		Philippines		Sri Lanka		Timor-Leste		Viet Nam		Mauritius	
	SDI	Rate	SDI	Rate	SDI	Rate	SDI	Rate	SDI	Rate	SDI	Rate
1990	0.507	140.3293	0.510	161.9071	0.523	150.0371	0.262	164.6927	0.408	158.4214	0.545	151.2972
1991	0.516	140.1309	0.514	161.9508	0.528	149.8264	0.271	164.4050	0.413	158.5721	0.548	151.0660
1992	0.525	139.7346	0.517	161.9330	0.533	149.5379	0.280	164.0327	0.420	158.4589	0.553	151.0858
1993	0.534	139.3817	0.521	161.9533	0.539	148.9594	0.289	163.4884	0.427	158.4535	0.561	151.0826
1994	0.543	139.0707	0.525	161.9079	0.545	148.5625	0.299	163.1480	0.435	158.5078	0.569	150.5860
1995	0.552	138.9011	0.529	161.8936	0.552	148.2167	0.308	162.7050	0.444	158.5798	0.577	150.5907
1996	0.561	138.5491	0.533	161.7926	0.559	147.6050	0.318	162.1042	0.453	158.4143	0.585	150.2139
1997	0.568	138.0272	0.537	161.6219	0.566	146.9725	0.327	161.3027	0.463	158.1343	0.591	149.8701
1998	0.574	137.7521	0.539	161.4544	0.572	146.0654	0.335	160.5178	0.472	157.8332	0.597	149.2938
1999	0.579	137.5041	0.542	161.2386	0.578	145.2484	0.339	159.9033	0.481	157.5678	0.602	148.6623
2000	0.584	137.5603	0.545	161.0752	0.583	144.6994	0.344	159.3357	0.490	157.3855	0.607	148.4206
2001	0.588	138.4095	0.547	160.8701	0.588	144.3818	0.349	158.9042	0.498	156.9521	0.612	148.3072
2002	0.592	140.5475	0.550	160.5741	0.592	144.0402	0.354	158.9594	0.505	156.4899	0.617	148.1077
2003	0.596	143.2656	0.552	160.2716	0.597	143.9181	0.358	158.9771	0.513	155.8594	0.623	147.9163
2004	0.601	145.5712	0.554	159.9311	0.602	143.5738	0.363	159.2302	0.521	155.3974	0.629	147.8828
2005	0.605	146.6737	0.556	159.5782	0.607	142.9543	0.367	159.0670	0.529	155.0215	0.635	147.9318
2006	0.610	147.3015	0.558	159.1882	0.612	142.5495	0.371	158.9254	0.536	154.5965	0.640	148.3704
2007	0.616	147.5695	0.561	158.6295	0.617	142.4105	0.375	158.7087	0.543	154.2308	0.644	148.9022
2008	0.621	147.5878	0.564	157.9951	0.622	142.2850	0.381	158.6763	0.550	153.9534	0.650	149.5199
2009	0.624	148.1108	0.567	157.3551	0.628	141.9203	0.388	158.5837	0.556	153.3968	0.655	150.2818
2010	0.629	147.9488	0.572	156.8884	0.634	141.7504	0.395	158.3234	0.563	152.9910	0.660	150.4302
2011	0.633	147.6584	0.577	156.4511	0.641	141.5386	0.402	158.1646	0.569	152.7058	0.666	150.5651
2012	0.638	146.9896	0.584	155.9304	0.648	141.1918	0.409	157.9122	0.575	152.4400	0.673	150.6026
2013	0.644	146.3932	0.591	155.3619	0.655	140.8733	0.415	157.7326	0.581	152.3286	0.680	150.7031
2014	0.649	145.5246	0.598	154.8994	0.663	140.7723	0.421	157.3524	0.587	151.9056	0.687	150.5834
2015	0.654	145.1748	0.606	154.5829	0.670	140.7244	0.426	157.2805	0.593	151.8409	0.692	150.6183
2016	0.659	145.0974	0.614	154.2876	0.676	140.6665	0.431	156.8465	0.599	151.7083	0.697	150.6438
2017	0.665	145.3371	0.622	153.9210	0.682	140.8704	0.435	156.7539	0.605	151.6012	0.702	150.7123
2018	0.670	145.2273	0.630	153.5874	0.688	140.8440	0.437	156.4738	0.611	151.7319	0.706	150.7678
2019	0.675	145.3317	0.639	153.3482	0.694	140.9721	0.440	156.3287	0.618	151.4389	0.711	151.1097
2020	0.679	144.7039	0.645	153.2362	0.698	140.8714	0.442	156.0983	0.623	150.9615	0.715	150.9798
2021	0.683	142.2941	0.651	152.8231	0.702	140.8659	0.445	155.5929	0.628	150.7022	0.718	150.7388

Table S18 (continued).												
Year	Seychelles		High-income Asia Pacific		Singapore		Japan		Republic of Korea		Brunei Darussalam	
	SDI	Rate	SDI	Rate	SDI	Rate	SDI	Rate	SDI	Rate	SDI	Rate
1990	0.576	156.2688	0.768	156.1569	0.686	120.7344	0.790	154.4951	0.692	173.4777	0.666	135.0958
1991	0.583	156.2734	0.773	156.2155	0.695	120.6315	0.795	154.4500	0.703	173.7231	0.671	135.0797
1992	0.590	155.8969	0.779	156.4140	0.703	120.5127	0.799	154.5339	0.712	174.2220	0.677	134.7021
1993	0.598	155.9083	0.784	156.6280	0.712	120.1855	0.803	154.6894	0.722	174.4102	0.682	134.4319
1994	0.606	155.7198	0.789	156.9919	0.721	120.0109	0.807	154.9720	0.732	174.9498	0.687	134.3004
1995	0.614	155.3627	0.794	157.2739	0.730	120.1008	0.811	155.2188	0.742	175.2492	0.692	134.0873
1996	0.622	155.2678	0.799	157.8855	0.739	119.9159	0.814	155.8298	0.752	175.6701	0.698	133.7863
1997	0.631	154.8551	0.803	158.8353	0.748	119.8239	0.817	156.8171	0.761	176.0894	0.703	133.4599
1998	0.638	154.2818	0.807	160.0636	0.755	119.5790	0.819	158.1595	0.768	176.4176	0.709	132.9870
1999	0.645	153.9521	0.810	161.3771	0.761	119.4928	0.821	159.6122	0.777	176.6359	0.715	132.6062
2000	0.652	153.7079	0.814	162.5904	0.768	119.4649	0.823	160.9484	0.785	176.9072	0.722	132.3665
2001	0.656	153.1873	0.818	164.0548	0.774	119.8001	0.825	162.5620	0.793	177.0184	0.729	132.5514
2002	0.661	152.8676	0.821	165.8335	0.780	120.5341	0.827	164.4778	0.801	177.3543	0.735	132.8178
2003	0.664	152.3798	0.825	167.6673	0.784	121.3824	0.829	166.4503	0.808	177.7574	0.742	132.8768
2004	0.666	151.6721	0.828	168.9955	0.790	121.8366	0.832	167.9024	0.814	177.8828	0.747	133.3479
2005	0.669	151.4867	0.831	169.6634	0.796	122.2571	0.834	168.5573	0.819	178.3354	0.752	133.6125
2006	0.672	151.2722	0.834	169.2984	0.803	122.3188	0.836	168.0727	0.824	178.6073	0.757	133.6844
2007	0.675	150.9338	0.837	168.2560	0.811	122.0946	0.838	166.7934	0.829	179.0643	0.762	134.0040
2008	0.677	150.8013	0.839	167.0290	0.818	121.9255	0.840	165.3032	0.834	179.6016	0.766	134.1251
2009	0.678	150.7613	0.842	166.0003	0.824	121.5618	0.842	164.0305	0.838	180.0292	0.770	134.2822
2010	0.680	150.4469	0.845	165.6800	0.830	121.5586	0.844	163.5452	0.843	180.4203	0.773	134.3375
2011	0.684	150.3571	0.848	166.3861	0.834	121.5141	0.847	164.3521	0.847	180.3768	0.777	134.4770
2012	0.688	150.0956	0.851	168.0281	0.837	121.4509	0.849	166.3040	0.851	180.4248	0.780	134.5850
2013	0.693	149.6381	0.854	169.9368	0.840	121.3298	0.851	168.5775	0.855	180.4493	0.784	134.7925
2014	0.697	149.4398	0.857	171.5467	0.843	121.1164	0.854	170.5381	0.860	180.2400	0.788	134.9147
2015	0.702	149.1622	0.860	172.3206	0.845	121.2311	0.856	171.4876	0.864	180.0199	0.791	134.8537
2016	0.706	149.2116	0.863	172.3859	0.848	122.2744	0.859	171.6663	0.868	178.8741	0.795	134.6183
2017	0.711	149.0865	0.866	172.2413	0.849	124.2054	0.861	171.8610	0.873	176.0833	0.798	134.2471
2018	0.716	148.8809	0.869	171.9832	0.851	126.2999	0.864	171.9450	0.877	173.1309	0.801	134.0975
2019	0.722	148.8506	0.872	171.8576	0.852	127.3241	0.867	171.9357	0.881	171.8141	0.804	133.6782
2020	0.727	149.1025	0.874	171.5461	0.854	127.4185	0.869	171.5356	0.884	171.8582	0.807	133.7345
2021	0.730	148.7386	0.877	165.4981	0.856	126.8607	0.871	164.2901	0.887	171.1852	0.810	132.9791

Table S19. Risk Factors for Alzheimer’s Disease and Other Dementias in Both Sexes in Non-High-income East Asia , 2021									
Age	Smoking			High fasting plasma glucose			High body-mass index		
	Percentage (%)	Number	Rate (per 100k)	Percentage (%)	Number	Rate (per 100k)	Percentage (%)	Number	Rate (per 100k)
40-44	10.17%(6.76%,14.49%)	345 (123,762)	0.36(0.13,0.8)	6.31%(0.58%,12.22%)	215 (20,523)	0.23(0.02,0.55)	5.94%(-0.78%,17.33%)	202 (-31,652)	0.21(-0.03,0.68)
45-49	10.48%(6.87%,14.59%)	2789 (1323,5036)	2.45(1.16,4.42)	7.51%(0.69%,14.67%)	2003 (153,4595)	1.76(0.13,4.03)	6.33%(-1.11%,18.46%)	1691 (-236,5530)	1.48(-0.21,4.85)
50-54	11.05%(7.55%,14.82%)	8632 (4603,13764)	6.92(3.69,11.03)	8.85%(0.77%,17.88%)	6962 (481,15657)	5.58(0.39,12.55)	6.71%(-1.14%,19.75%)	5278 (-721,16329)	4.23(-0.58,13.08)
55-59	10.57%(7.19%,14.32%)	16471 (9120,25846)	14.52(8.04,22.79)	9.88%(0.85%,19.46%)	15385 (1098,32709)	13.56(0.97,28.84)	6.4%(-1.2%,18.49%)	9906 (-1855,31247)	8.73(-1.64,27.55)
60-64	9.59%(6.59%,12.72%)	19918 (11021,32078)	26.2(14.5,42.2)	11.08%(0.96%,21.75%)	22900 (1760,48359)	30.13(2.32,63.62)	6.46%(-1.21%,18.62%)	13305 (-2624,39320)	17.5(-3.45,51.73)
65-69	8.71%(6.1%,11.62%)	34200 (19823,54412)	43.23(25.06,68.78)	11.97%(1.04%,23.47%)	47034 (4007,101318)	59.46(5.07,128.08)	6.24%(-1.04%,17.92%)	24474 (-4093,73888)	30.94(-5.17,93.4)
70-74	6.95%(4.82%,9.32%)	35068 (19009,55655)	63.79(34.58,101.24)	12.6%(1.05%,24.96%)	63655 (5141,141059)	115.79(9.35,256.59)	5.55%(-0.7%,16.41%)	27951 (-3934,90900)	50.84(-7.16,165.35)
75-79	5.74%(3.97%,7.77%)	34385 (19517,56561)	100.33(56.95,165.04)	12.74%(1.13%,25.19%)	76259 (6633,167860)	222.52(19.36,489.8)	4.71%(-0.52%,13.54%)	28168 (-3197,90907)	82.19(-9.33,265.26)
80-84	4.2%(2.72%,5.63%)	29857 (16676,46481)	144.87(80.91,225.53)	12.52%(1.08%,25.45%)	89079 (8077,199720)	432.22(39.19,969.07)	3.28%(-0.15%,9.67%)	23382 (-984,78120)	113.45(-4.77,379.05)
85-89	3.73%(2.4%,5.07%)	20922 (12077,32934)	210.79(121.68,331.82)	11.97%(0.96%,24.3%)	67264 (5581,147833)	677.7(56.23,1489.46)	3.39%(-0.19%,10.09%)	19141 (-1026,62575)	192.85(-10.33,630.46)
90-94	3.14%(2.02%,4.26%)	7604 (4229,12276)	245.6(136.59,396.52)	11.22%(0.96%,22.36%)	27253 (2361,58194)	880.27(76.25,1879.68)	3.5%(-0.24%,10.38%)	8546 (-493,28615)	276.03(-15.94,924.25)
95+	2.43%(1.49%,3.48%)	1657 (918,2641)	235.74(130.64,375.74)	10.23%(0.88%,20.68%)	6969 (671,15407)	991.52(95.43,2191.98)	3.59%(-0.27%,10.86%)	2442 (-166,7977)	347.37(-23.69,1134.81)

Table S20. Risk Factors for Alzheimer’s Disease and Other Dementias in Male Sexes in Non-High-income East Asia , 2021									
Age	Smoking			High fasting plasma glucose			High body-mass index		
	Percentage (%)	Number	Rate (per 100k)	Percentage (%)	Number	Rate (per 100k)	Percentage (%)	Number	Rate (per 100k)
40-44	21.17%(15.02%,27.54%)	320 (110,713)	0.65(0.22,1.46)	7.05%(0.68%,14.06%)	107 (10,282)	0.22(0.02,0.58)	5.62%(-0.69%,16.73%)	85 (-13,289)	0.17(-0.03,0.59)
45-49	21.69%(15.35%,28.02%)	2605 (1239,4722)	4.5(2.14,8.15)	8.27%(0.76%,16.46%)	997 (79,2476)	1.72(0.14,4.27)	5.5%(-0.73%,16.54%)	660 (-68,2217)	1.14(-0.12,3.83)
50-54	22.59%(16.43%,29%)	7912 (4211,12606)	12.54(6.68,19.98)	9.42%(0.82%,19.12%)	3307 (221,7728)	5.24(0.35,12.25)	5.29%(-0.51%,15.82%)	1861 (-150,5816)	2.95(-0.24,9.22)
55-59	21.51%(15.45%,27.87%)	14708 (8087,23276)	26(14.3,41.14)	10.13%(0.86%,20.45%)	6923 (457,15061)	12.24(0.81,26.62)	4.73%(-0.37%,14.19%)	3209 (-220,10426)	5.67(-0.39,18.43)
60-64	19.59%(13.63%,25.47%)	17799 (9803,28197)	46.77(25.76,74.1)	11.13%(0.98%,22.08%)	10059 (752,21135)	26.43(1.98,55.54)	4.67%(-0.38%,14.15%)	4201 (-333,13701)	11.04(-0.87,36.01)
65-69	18.4%(13.23%,23.79%)	29491 (17181,46668)	75.92(44.23,120.15)	11.88%(1.1%,23.54%)	19063 (1703,41620)	49.08(4.39,107.15)	4.16%(-0.35%,12.5%)	6656 (-601,20243)	17.14(-1.55,52.11)
70-74	15.77%(11%,20.71%)	29370 (15758,45676)	110.47(59.27,171.79)	12.61%(1.03%,25.46%)	23529 (1828,52876)	88.5(6.88,198.87)	3.46%(-0.23%,10.37%)	6437 (-472,20007)	24.21(-1.77,75.25)
75-79	13.58%(9.55%,17.82%)	28091 (15657,45851)	175.13(97.61,285.86)	12.91%(1.12%,25.59%)	26707 (2401,58220)	166.5(14.97,362.97)	3.1%(-0.1%,9.31%)	6403 (-223,21294)	39.92(-1.39,132.76)
80-84	9.89%(6.8%,13.23%)	22902 (12744,37000)	255.38(142.11,412.6)	12.81%(1.19%,26.53%)	29613 (2989,67504)	330.22(33.33,752.75)	2.59%(-0.04%,8.04%)	5996 (-107,19757)	66.86(-1.19,220.31)
85-89	9.67%(6.6%,12.96%)	15100 (8871,23951)	418.24(245.72,663.38)	12.2%(0.94%,25.27%)	19027 (1525,41637)	527.01(42.23,1153.25)	2.64%(-0.05%,8.13%)	4132 (-80,13113)	114.44(-2.2,363.19)
90-94	9.44%(6.44%,12.72%)	4875 (2774,8069)	560.43(318.82,927.49)	11.19%(0.98%,22.2%)	5793 (480,12623)	665.9(55.18,1450.96)	2.65%(-0.05%,8.27%)	1377 (-25,4777)	158.26(-2.91,549.14)
95+	8.71%(5.95%,11.63%)	889 (516,1436)	595.73(346.15,962.71)	10.19%(0.87%,20.31%)	1036 (92,2229)	694.65(61.57,1494.41)	2.76%(-0.06%,8.72%)	280 (-6,927)	187.91(-3.98,621.47)

Table S21. Risk Factors for Alzheimer’ s Disease and Other Dementias in Female Sexes in Non-High-income East Asia , 2021									
Age	Smoking			High fasting plasma glucose			High body-mass index		
	Percentage (%)	Number	Rate (per 100k)	Percentage (%)	Number	Rate (per 100k)	Percentage (%)	Number	Rate (per 100k)
40-44	1.37%(0.76%,2.44%)	26 (8,61)	0.06(0.02,0.13)	5.72%(0.47%,11.66%)	108 (10,297)	0.23(0.02,0.64)	6.19%(-0.95%,18.3%)	117 (-18,372)	0.25(-0.04,0.8)
45-49	1.26%(0.65%,2.35%)	184 (62,392)	0.33(0.11,0.7)	6.88%(0.62%,14.22%)	1007 (78,2532)	1.79(0.14,4.51)	7.01%(-1.48%,20.61%)	1031 (-169,3332)	1.84(-0.3,5.94)
50-54	1.67%(0.82%,3.03%)	720 (275,1418)	1.17(0.44,2.3)	8.39%(0.73%,16.93%)	3656 (259,8584)	5.92(0.42,13.91)	7.86%(-1.66%,22.78%)	3417 (-610,10692)	5.54(-0.99,17.32)
55-59	2.02%(1.04%,3.7%)	1763 (796,3488)	3.1(1.4,6.14)	9.68%(0.84%,19.36%)	8462 (641,18432)	14.88(1.13,32.42)	7.71%(-1.88%,22.25%)	6698 (-1481,20527)	11.78(-2.61,36.11)
60-64	1.81%(0.93%,3.21%)	2119 (882,4099)	5.58(2.32,10.8)	11.05%(0.95%,21.62%)	12842 (951,27800)	33.83(2.51,73.24)	7.85%(-1.86%,22.22%)	9105 (-2036,26517)	23.99(-5.36,69.86)
65-69	2.03%(1.05%,3.56%)	4710 (1923,8977)	11.7(4.77,22.29)	12.03%(1%,23.36%)	27970 (2245,61430)	69.46(5.58,152.56)	7.67%(-1.74%,21.32%)	17818 (-3776,54343)	44.25(-9.38,134.96)
70-74	1.79%(0.92%,3.05%)	5698 (2478,11422)	20.07(8.73,40.24)	12.58%(1.06%,24.55%)	40125 (3312,90961)	141.35(11.67,320.43)	6.76%(-1.08%,19.89%)	21515 (-4029,66980)	75.79(-14.19,235.95)
75-79	1.61%(0.81%,2.88%)	6294 (2894,12393)	34.52(15.87,67.98)	12.64%(1.14%,25.05%)	49551 (4233,108740)	271.8(23.22,596.45)	5.56%(-0.79%,15.75%)	21765 (-2927,69192)	119.39(-16.06,379.53)
80-84	1.46%(0.73%,2.41%)	6955 (3320,12350)	59.74(28.52,106.08)	12.38%(1.02%,25.12%)	59466 (5076,133365)	510.8(43.6,1145.56)	3.61%(-0.25%,10.55%)	17387 (-1110,58184)	149.35(-9.53,499.78)
85-89	1.44%(0.72%,2.38%)	5821 (2729,10117)	92.19(43.21,160.2)	11.88%(0.96%,23.87%)	48237 (4057,104676)	763.85(64.24,1657.6)	3.68%(-0.28%,10.97%)	15009 (-1104,51121)	237.68(-17.48,809.53)
90-94	1.44%(0.71%,2.37%)	2728 (1293,4776)	122.56(58.1,214.55)	11.22%(0.96%,22.34%)	21460 (1852,45245)	964.05(83.19,2032.53)	3.73%(-0.31%,11.28%)	7169 (-510,24566)	322.05(-22.92,1103.57)
95+	1.33%(0.64%,2.26%)	768 (361,1356)	138.77(65.26,244.83)	10.24%(0.88%,20.72%)	5933 (579,13209)	1071.49(104.56,2385.45)	3.74%(-0.32%,11.51%)	2161 (-167,7318)	390.33(-30.19,1321.53)

Table S22. Risk Factors for Alzheimer’ s Disease and Other Dementias in Both Sexes in Non-High-income Southeast Asia , 2021									
Age	Smoking			High fasting plasma glucose			High body-mass index		
	Percentage (%)	Number	Rate (per 100k)	Percentage (%)	Number	Rate (per 100k)	Percentage (%)	Number	Rate (per 100k)
40-44	7.73%(4.96%,10.95%)	136 (48,276)	0.28(0.1,0.56)	3.8%(0.32%,7.65%)	67 (6,175)	0.14(0.01,0.36)	5.05%(-0.77%,14.27%)	90 (-11,298)	0.18(-0.02,0.61)
45-49	7.85%(5.31%,10.78%)	798 (384,1403)	1.8(0.86,3.16)	5.16%(0.43%,10.24%)	527 (39,1238)	1.19(0.09,2.79)	5.14%(-0.83%,13.75%)	527 (-58,1709)	1.19(-0.13,3.85)
50-54	7.76%(5.43%,10.43%)	1865 (979,3007)	4.63(2.43,7.46)	6.83%(0.57%,13.74%)	1651 (108,3680)	4.1(0.27,9.13)	5.12%(-0.73%,14.05%)	1242 (-133,3779)	3.08(-0.33,9.38)
55-59	7.16%(5%,9.52%)	3083 (1759,4847)	8.85(5.05,13.91)	8.59%(0.73%,17.34%)	3690 (260,8291)	10.59(0.75,23.8)	4.86%(-0.7%,13.83%)	2083 (-257,6244)	5.98(-0.74,17.92)
60-64	6.44%(4.6%,8.58%)	4216 (2428,6842)	14.76(8.5,23.96)	11.11%(0.88%,22.93%)	7219 (496,15511)	25.28(1.74,54.32)	4.11%(-0.46%,11.76%)	2667 (-290,8108)	9.34(-1.01,28.4)
65-69	5.63%(3.98%,7.4%)	4667 (2616,7499)	22.14(12.41,35.58)	14.31%(1.06%,30.09%)	11866 (844,27231)	56.29(4,129.18)	3.77%(-0.37%,10.65%)	3134 (-332,9355)	14.87(-1.57,44.38)
70-74	4.49%(3.21%,5.81%)	4234 (2378,6486)	31.17(17.51,47.76)	15.97%(1.18%,33.7%)	15081 (1063,35169)	111.05(7.83,258.98)	3.34%(-0.3%,10.06%)	3166 (-326,10396)	23.31(-2.4,76.55)
75-79	3.63%(2.58%,4.75%)	3706 (2166,5797)	44.84(26.21,70.14)	16.48%(1.19%,34.97%)	16818 (1200,39572)	203.5(14.52,478.85)	2.86%(-0.21%,8.57%)	2919 (-195,9528)	35.32(-2.35,115.29)
80-84	3.11%(2.22%,4.08%)	3476 (2023,5421)	72.03(41.9,112.31)	16.84%(1.21%,35.88%)	18797 (1467,44087)	389.44(30.4,913.43)	1.52%(-0.11%,5.02%)	1698 (-131,6363)	35.18(-2.71,131.84)
85-89	2.87%(2.04%,3.82%)	2498 (1481,3949)	107.2(63.58,169.45)	16.79%(1.23%,36.38%)	14631 (1164,32929)	627.88(49.96,1413.14)	1.6%(-0.1%,5.34%)	1400 (-98,5308)	60.09(-4.19,227.8)
90-94	2.67%(1.89%,3.52%)	1169 (724,1862)	139.52(86.35,222.18)	15.5%(1.15%,33.03%)	6807 (510,15733)	812.27(60.8,1877.39)	1.73%(-0.1%,5.69%)	759 (-44,2632)	90.56(-5.27,314.02)
95+	2.44%(1.7%,3.24%)	382 (232,595)	159.74(96.95,249.1)	13.82%(1.09%,29.67%)	2154 (191,4853)	901.74(79.85,2031.71)	1.86%(-0.09%,6.25%)	290 (-15,1052)	121.36(-6.15,440.4)

Table S23. Risk Factors for Alzheimer’s Disease and Other Dementias in Male Sexes in Non-High-income Southeast Asia , 2021									
Age	Smoking			High fasting plasma glucose			High body-mass index		
	Percentage (%)	Number	Rate (per 100k)	Percentage (%)	Number	Rate (per 100k)	Percentage (%)	Number	Rate (per 100k)
40-44	16.14%(11.77%,20.37%)	126 (44,258)	0.51(0.18,1.05)	3.71%(0.3%,7.48%)	29 (2,82)	0.12(0.01,0.33)	3.44%(-0.34%,10.23%)	27 (-2,99)	0.11(-0.01,0.4)
45-49	16.15%(11.72%,20.37%)	730 (343,1276)	3.3(1.55,5.77)	4.98%(0.39%,10.09%)	227 (16,550)	1.03(0.07,2.49)	3.57%(-0.36%,10.15%)	164 (-12,529)	0.74(-0.06,2.39)
50-54	16.09%(11.73%,20.76%)	1686 (895,2740)	8.49(4.51,13.8)	6.49%(0.53%,13.15%)	682 (43,1557)	3.43(0.22,7.84)	3.39%(-0.34%,9.81%)	358 (-24,1194)	1.8(-0.12,6.02)
55-59	15.08%(10.89%,19.37%)	2722 (1538,4354)	16.08(9.09,25.72)	8.26%(0.7%,16.77%)	1489 (99,3407)	8.8(0.58,20.13)	3.24%(-0.26%,9.7%)	586 (-36,1814)	3.46(-0.21,10.72)
60-64	13.88%(10.05%,17.8%)	3683 (2091,6016)	26.97(15.32,44.07)	10.81%(0.85%,22.72%)	2846 (188,6040)	20.84(1.38,44.24)	2.42%(-0.07%,8.02%)	637 (-18,2128)	4.67(-0.13,15.59)
65-69	12.41%(8.99%,16.05%)	3986 (2219,6474)	40.56(22.58,65.88)	14.39%(1.05%,30.54%)	4620 (336,10576)	47.01(3.42,107.62)	1.77%(-0.12%,5.81%)	572 (-37,2057)	5.82(-0.38,20.93)
70-74	10.2%(7.25%,13.2%)	3493 (2000,5442)	56.76(32.5,88.43)	16.24%(1.21%,35.05%)	5563 (394,13065)	90.4(6.4,212.31)	1.49%(-0.13%,5.11%)	512 (-48,1999)	8.31(-0.78,32.49)
75-79	8.85%(6.38%,11.38%)	3000 (1749,4765)	85.08(49.59,135.13)	16.79%(1.22%,35.3%)	5689 (420,13231)	161.34(11.91,375.22)	1.12%(-0.19%,4.01%)	379 (-72,1493)	10.75(-2.04,42.34)
80-84	7.79%(5.51%,10.27%)	2674 (1568,4160)	140.36(82.33,218.41)	17.35%(1.23%,36.74%)	5938 (468,13718)	311.74(24.55,720.19)	0.57%(-0.55%,2.9%)	199 (-187,1082)	10.46(-9.79,56.83)
85-89	7.5%(5.3%,9.86%)	1895 (1142,3021)	221.35(133.38,352.86)	17.47%(1.27%,37.37%)	4422 (337,10003)	516.44(39.39,1168.15)	0.64%(-0.5%,3%)	163 (-131,823)	18.99(-15.35,96.07)
90-94	7.11%(5.04%,9.29%)	886 (537,1412)	296.43(179.76,472.18)	16.34%(1.23%,34.48%)	2045 (141,4761)	684.09(47.02,1592.63)	0.78%(-0.44%,3.37%)	98 (-61,464)	32.76(-20.48,155.27)
95+	6.32%(4.49%,8.43%)	298 (179,467)	334.16(201.54,524.15)	14.66%(1.06%,30.54%)	688 (51,1547)	772.91(57.5,1737.01)	1%(-0.36%,4.02%)	47 (-20,207)	53.05(-22.78,232.42)

Table S24. Risk Factors for Alzheimer’s Disease and Other Dementias in Female Sexes in Non-High-income Southeast Asia , 2021									
Age	Smoking			High fasting plasma glucose			High body-mass index		
	Percentage (%)	Number	Rate (per 100k)	Percentage (%)	Number	Rate (per 100k)	Percentage (%)	Number	Rate (per 100k)
40-44	1.05%(0.66%,1.52%)	10 (3,21)	0.04(0.01,0.08)	3.88%(0.33%,7.69%)	38 (3,99)	0.16(0.01,0.41)	6.32%(-1.12%,17.66%)	63 (-9,218)	0.26(-0.04,0.89)
45-49	1.2%(0.76%,1.8%)	68 (28,127)	0.3(0.13,0.57)	5.31%(0.47%,10.44%)	300 (24,701)	1.35(0.11,3.15)	6.41%(-1.2%,17.58%)	364 (-62,1215)	1.63(-0.28,5.45)
50-54	1.32%(0.89%,1.92%)	180 (88,304)	0.88(0.43,1.49)	7.09%(0.59%,14.11%)	969 (65,2229)	4.74(0.32,10.91)	6.45%(-1.13%,18.33%)	884 (-119,2705)	4.33(-0.58,13.24)
55-59	1.44%(0.95%,2.18%)	360 (193,603)	2.01(1.08,3.37)	8.84%(0.74%,17.9%)	2201 (162,4845)	12.29(0.9,27.04)	6.03%(-1.11%,17.29%)	1498 (-269,4489)	8.36(-1.5,25.06)
60-64	1.37%(0.88%,1.96%)	533 (292,920)	3.58(1.96,6.17)	11.32%(0.9%,23.02%)	4373 (309,9293)	29.35(2.07,62.36)	5.26%(-0.87%,15.24%)	2030 (-366,6017)	13.62(-2.46,40.38)
65-69	1.34%(0.87%,1.93%)	681 (371,1155)	6.05(3.29,10.26)	14.26%(1.06%,29.96%)	7246 (508,16904)	64.39(4.51,150.21)	5.03%(-0.72%,14.35%)	2563 (-420,7759)	22.77(-3.73,68.95)
70-74	1.23%(0.8%,1.79%)	741 (383,1255)	9.97(5.16,16.9)	15.82%(1.16%,33.44%)	9517 (669,21858)	128.16(9.01,294.35)	4.4%(-0.88%,13.5%)	2655 (-489,8793)	35.75(-6.59,118.41)
75-79	1.03%(0.7%,1.45%)	706 (377,1164)	14.9(7.97,24.57)	16.33%(1.18%,34.76%)	11128 (780,26029)	234.89(16.46,549.4)	3.72%(-0.49%,10.87%)	2540 (-301,8262)	53.61(-6.35,174.39)
80-84	1.04%(0.69%,1.49%)	803 (444,1301)	27.48(15.2,44.54)	16.62%(1.2%,35.58%)	12858 (978,30547)	440.1(33.46,1045.49)	1.93%(-0.11%,6.44%)	1499 (-83,5502)	51.3(-2.84,188.33)
85-89	0.97%(0.65%,1.4%)	603 (321,982)	40.88(21.81,66.64)	16.51%(1.21%,35.3%)	10209 (813,23096)	692.62(55.19,1566.93)	2%(-0.11%,6.84%)	1238 (-65,4512)	83.97(-4.42,306.09)
90-94	0.91%(0.6%,1.3%)	283 (163,471)	52.51(30.16,87.43)	15.17%(1.12%,32.28%)	4762 (369,10694)	883.34(68.44,1983.78)	2.11%(-0.1%,7.3%)	661 (-32,2234)	122.61(-5.93,414.42)
95+	0.77%(0.49%,1.16%)	84 (46,140)	56.03(30.97,93.13)	13.46%(1.1%,28.49%)	1466 (130,3272)	978.34(86.64,2184.44)	2.23%(-0.11%,7.72%)	243 (-12,922)	161.98(-8.15,615.15)

Table S25. Risk Factors for Alzheimer’s Disease and Other Dementias in Both Sexes in High-income Asia Pacific , 2021									
Age	Smoking			High fasting plasma glucose			High body-mass index		
	Percentage (%)	Number	Rate (per 100k)	Percentage (%)	Number	Rate (per 100k)	Percentage (%)	Number	Rate (per 100k)
40-44	9.58%(6.47%,13.25%)	37 (13,79)	0.29(0.1,0.6)	5.82%(0.49%,11.8%)	23 (2,59)	0.18(0.02,0.45)	3.46%(-0.2%,11.02%)	14 (-1,46)	0.1(-0.01,0.35)
45-49	8.82%(5.78%,12.12%)	277 (126,508)	1.9(0.86,3.48)	6.89%(0.6%,13.46%)	218 (15,503)	1.49(0.11,3.44)	3.7%(-0.2%,11.11%)	118 (-7,391)	0.81(-0.05,2.68)
50-54	8.31%(5.65%,11.29%)	656 (342,1080)	4.59(2.39,7.56)	8.25%(0.69%,15.62%)	656 (43,1480)	4.59(0.3,10.35)	3.8%(-0.19%,11.91%)	302 (-10,999)	2.11(-0.07,6.99)
55-59	8.11%(5.4%,11.18%)	1126 (605,1772)	9.03(4.85,14.2)	9.81%(0.88%,18.53%)	1361 (105,2844)	10.9(0.84,22.79)	3.99%(-0.37%,12.35%)	552 (-37,1750)	4.43(-0.29,14.02)
60-64	6.34%(4.18%,8.66%)	1609 (875,2601)	13.54(7.36,21.88)	11.6%(1.04%,22.25%)	2931 (236,5935)	24.66(1.99,49.93)	3.97%(-0.34%,12.25%)	1001 (-67,3215)	8.42(-0.56,27.05)
65-69	6.31%(4.41%,8.63%)	2750 (1569,4338)	24.56(14.01,38.73)	13.24%(1.15%,24.99%)	5793 (475,12244)	51.73(4.24,109.33)	3.84%(-0.44%,11.7%)	1682 (-178,5346)	15.02(-1.59,47.73)
70-74	4.77%(3.18%,6.6%)	4254 (2320,6758)	35.22(19.21,55.95)	14.14%(1.26%,27.6%)	12650 (1045,26607)	104.73(8.65,220.28)	3.25%(-0.07%,10.01%)	2915 (-61,10113)	24.13(-0.51,83.73)
75-79	3.39%(2.31%,4.81%)	3983 (2342,6391)	47.13(27.71,75.61)	15.28%(1.38%,29.04%)	17921 (1576,37642)	212.03(18.65,445.34)	3.09%(-0.27%,10.15%)	3631 (-254,12901)	42.96(-3,152.64)
80-84	2.75%(1.85%,3.86%)	4956 (2911,8126)	73.21(43,120.05)	16.34%(1.46%,31.14%)	29435 (2686,61622)	434.82(39.68,910.29)	2.08%(-0.09%,6.83%)	3759 (-154,12949)	55.52(-2.28,191.28)
85-89	2.25%(1.49%,3.18%)	4346 (2425,6987)	96.56(53.89,155.26)	16.38%(1.37%,31.58%)	31633 (2843,66825)	702.9(63.18,1484.89)	2.11%(-0.07%,7.05%)	4087 (-122,14778)	90.83(-2.71,328.38)
90-94	1.81%(1.18%,2.59%)	2368 (1329,4066)	107.84(60.52,185.18)	13.88%(1.23%,26.63%)	18239 (1568,38456)	830.57(71.39,1751.22)	2.12%(-0.09%,7.26%)	2814 (-116,9953)	128.17(-5.3,453.24)
95+	1.38%(0.86%,2.04%)	985 (537,1623)	104.54(57.04,172.25)	13.14%(1.23%,25.93%)	9368 (946,20868)	994.2(100.39,2214.8)	2.11%(-0.11%,7.26%)	1501 (-74,5405)	159.29(-7.88,573.6)

Table S26. Risk Factors for Alzheimer’s Disease and Other Dementias in Male Sexes in High-income Asia Pacific , 2021									
Age	Smoking			High fasting plasma glucose			High body-mass index		
	Percentage (%)	Number	Rate (per 100k)	Percentage (%)	Number	Rate (per 100k)	Percentage (%)	Number	Rate (per 100k)
40-44	15.95%(10.91%,20.68%)	29 (10,64)	0.44(0.15,0.96)	6.65%(0.55%,13.34%)	12 (1,34)	0.18(0.02,0.5)	3.92%(-0.17%,12.74%)	7 (0,25)	0.11(0,0.37)
45-49	15.14%(10.55%,20.42%)	213 (93,404)	2.87(1.25,5.43)	7.83%(0.66%,15.61%)	111 (8,268)	1.49(0.11,3.6)	4.45%(-0.23%,12.79%)	63 (-4,220)	0.85(-0.05,2.96)
50-54	15.24%(10.53%,19.95%)	511 (255,858)	7.09(3.53,11.89)	9.62%(0.8%,18.68%)	323 (20,705)	4.48(0.28,9.77)	4.15%(-0.4%,12.7%)	140 (-8,469)	1.93(-0.11,6.5)
55-59	14.77%(10.27%,19.57%)	854 (472,1352)	13.64(7.54,21.6)	11.21%(1%,21.71%)	646 (47,1424)	10.31(0.74,22.75)	4.03%(-0.22%,12.54%)	232 (-11,764)	3.7(-0.17,12.2)
60-64	11.89%(8.01%,15.94%)	1227 (650,2010)	20.88(11.06,34.19)	13.12%(1.16%,24.83%)	1347 (101,2861)	22.9(1.71,48.66)	3.28%(-0.11%,11.07%)	334 (-10,1183)	5.68(-0.18,20.12)
65-69	11.13%(7.78%,15.14%)	1940 (1089,3077)	35.61(19.99,56.48)	15.27%(1.36%,29.85%)	2669 (231,5428)	49(4.24,99.64)	2.79%(-0.11%,9.47%)	486 (-20,1570)	8.92(-0.37,28.82)
70-74	8.76%(5.9%,11.94%)	3017 (1683,4686)	52.8(29.44,82)	16.11%(1.44%,31.35%)	5566 (446,11938)	97.4(7.8,208.9)	2.26%(-0.1%,7.81%)	779 (-35,2821)	13.62(-0.61,49.37)
75-79	8.03%(5.52%,10.91%)	3321 (1980,5446)	87.98(52.45,144.28)	17.44%(1.5%,33.31%)	7199 (645,15602)	190.72(17.09,413.32)	1.95%(-0.16%,7.05%)	804 (-66,3096)	21.3(-1.74,82.02)
80-84	6.05%(4.09%,8.46%)	3424 (1955,5577)	122.7(70.06,199.86)	18.22%(1.61%,35.45%)	10296 (970,21512)	368.99(34.76,770.97)	1.22%(-0.44%,5.18%)	691 (-230,3089)	24.76(-8.23,110.7)
85-89	5.52%(3.69%,7.82%)	2787 (1589,4557)	173.49(98.95,283.68)	19.03%(1.57%,36.72%)	9607 (848,20505)	598.1(52.76,1276.54)	1.24%(-0.42%,5.32%)	625 (-195,2726)	38.89(-12.13,169.71)
90-94	5.03%(3.32%,7.22%)	1370 (747,2279)	212.15(115.65,352.92)	15.83%(1.4%,31.56%)	4323 (358,9560)	669.58(55.5,1480.57)	1.26%(-0.43%,5.37%)	342 (-109,1527)	53.02(-16.85,236.49)
95+	4.25%(2.7%,6.22%)	541 (291,930)	227.15(122.01,390.63)	15.21%(1.37%,29.38%)	1930 (180,4112)	810.4(75.76,1726.68)	1.28%(-0.43%,5.42%)	161 (-48,731)	67.65(-20.07,306.82)

Table S27. Risk Factors for Alzheimer' s Disease and Other Dementias in Female Sexes in High-income Asia Pacific , 2021									
Age	Smoking			High fasting plasma glucose			High body-mass index		
	Percentage (%)	Number	Rate (per 100k)	Percentage (%)	Number	Rate (per 100k)	Percentage (%)	Number	Rate (per 100k)
40-44	3.88%(2.25%,6.02%)	8 (2,18)	0.13(0.04,0.28)	5.08%(0.43%,10.42%)	11 (1,29)	0.17(0.01,0.45)	3.05%(-0.32%,10.71%)	6 (-1,24)	0.1(-0.01,0.38)
45-49	3.71%(2.06%,5.98%)	64 (26,126)	0.9(0.36,1.76)	6.12%(0.54%,12.27%)	107 (7,258)	1.49(0.1,3.6)	3.1%(-0.19%,10.59%)	54 (-4,212)	0.76(-0.05,2.95)
50-54	3.19%(1.9%,4.97%)	145 (66,268)	2.05(0.93,3.79)	7.25%(0.61%,14.01%)	333 (23,745)	4.7(0.32,10.51)	3.54%(-0.21%,11.31%)	163 (-10,579)	2.3(-0.15,8.18)
55-59	3.36%(1.87%,5.28%)	273 (117,470)	4.39(1.88,7.55)	8.82%(0.8%,17.05%)	715 (58,1462)	11.5(0.93,23.51)	3.95%(-0.47%,12.06%)	321 (-30,1050)	5.16(-0.48,16.89)
60-64	2.53%(1.47%,3.94%)	382 (181,686)	6.36(3.02,11.42)	10.55%(0.97%,20.03%)	1585 (124,3468)	26.38(2.06,57.74)	4.44%(-0.46%,13.4%)	668 (-60,2211)	11.11(-1,36.8)
65-69	3.1%(1.81%,4.94%)	810 (399,1398)	14.09(6.93,24.3)	11.89%(1%,22.91%)	3124 (243,6707)	54.32(4.22,116.61)	4.55%(-0.61%,13.08%)	1196 (-141,3777)	20.79(-2.45,65.68)
70-74	2.26%(1.25%,3.71%)	1237 (596,2244)	19.44(9.37,35.25)	12.9%(1.15%,25.87%)	7084 (588,14881)	111.3(9.23,233.84)	3.87%(-0.24%,11.37%)	2136 (-142,7569)	33.57(-2.24,118.93)
75-79	0.87%(0.54%,1.36%)	662 (328,1155)	14.16(7.02,24.7)	14.11%(1.3%,26.82%)	10722 (909,23596)	229.23(19.44,504.45)	3.72%(-0.42%,11.95%)	2827 (-290,9763)	60.43(-6.19,208.72)
80-84	1.24%(0.74%,1.95%)	1532 (770,2662)	38.51(19.35,66.9)	15.49%(1.38%,29.83%)	19140 (1685,41754)	480.99(42.35,1049.29)	2.48%(-0.1%,7.69%)	3068 (-94,10126)	77.09(-2.36,254.47)
85-89	1.1%(0.65%,1.74%)	1559 (796,2703)	53.87(27.5,93.39)	15.43%(1.31%,30.56%)	22026 (1996,46959)	761.07(68.96,1622.6)	2.42%(-0.11%,7.67%)	3463 (-146,12124)	119.65(-5.03,418.95)
90-94	0.96%(0.56%,1.58%)	998 (492,1896)	64.39(31.71,122.3)	13.37%(1.19%,26.34%)	13916 (1203,29316)	897.63(77.61,1891.05)	2.35%(-0.13%,7.76%)	2472 (-125,8823)	159.47(-8.05,569.12)
95+	0.76%(0.41%,1.34%)	444 (209,854)	63.06(29.71,121.3)	12.69%(1.21%,26.21%)	7437 (743,16814)	1056.38(105.51,2388.2)	2.29%(-0.14%,7.8%)	1340 (-88,4711)	190.28(-12.43,669.12)
